# Supplementary material for: Effects of climate and forest development on habitat specialization and biodiversity in Central European mountain forests
Source: Commun Biol. 2024 Nov 15;7:1518. doi: 10.1038/s42003-024-07239-6 (PMC11568152; doi:10.1038/s42003-024-07239-6)
Supplement: Supplementary file 2 — Supplementary Information [file 42003_2024_7239_MOESM2_ESM.pdf]

Supplementary material

Effects of climate and forest development on habitat specialization and biodiversity in Central European mountain forests

Tobias Richter[1,2], Lisa Geres[1,2,3], Sebastian König[1,2], Kristin H. Braziunas[1], Cornelius Senf[4], Dominik Thom[1,5,6], Claus Bässler[7,8], Jörg Müller[8,9], Rupert Seidl[1,2], and Sebastian Seibold[1,2,10]

Affiliations:

1 Technical University of Munich, School of Life Sciences, Ecosystem Dynamics and Forest Management Group, Hans-Carl-von-Carlowitz-Platz 2, 85354, Freising, Germany

2 Berchtesgaden National Park, Doktorberg 6, 83471, Berchtesgaden, Germany

3 Goethe University Frankfurt, Faculty of Biological Sciences, Institute for Ecology, Evolution and Diversity, Conservation Biology, 60438 Frankfurt am Main, Germany

4 Technical University of Munich, School of Life Sciences, Earth Observation for Ecosystem Management, Hans-Carl-von-Carlowitz-Platz 2, 85354 Freising, Germany

5 Chair of Silviculture, Institute of Silviculture and Forest Protection, TUD Dresden University of Technology, Pienner Str. 7, 01737 Tharandt, Germany

6 Gund Institute for Environment, University of Vermont, 617 Main Street, Burlington, VT 05405, USA

7 Ecology of Fungi, Bayreuth Center of Ecology and Environmental Research (BayCEER), University of Bayreuth, Universitätsstr. 30m, 95440 Bayreuth, Germany

8 Bavarian Forest National Park, Freyunger Strasse 2, 94481, Grafenau, Germany

9 Ecological Field Station Fabrikschleichach, Department of Animal Ecology and Tropical Biology, University of Würzburg, Glashüttenstraße 5, 96181, Rauhenebrach, Germany

10 Forest Zoology, Institute for Forest Botany and Forest Zoology, TUD Dresden University of Technology, Pienner Str. 7, 01737 Tharandt, Germany

Methods

Study Area

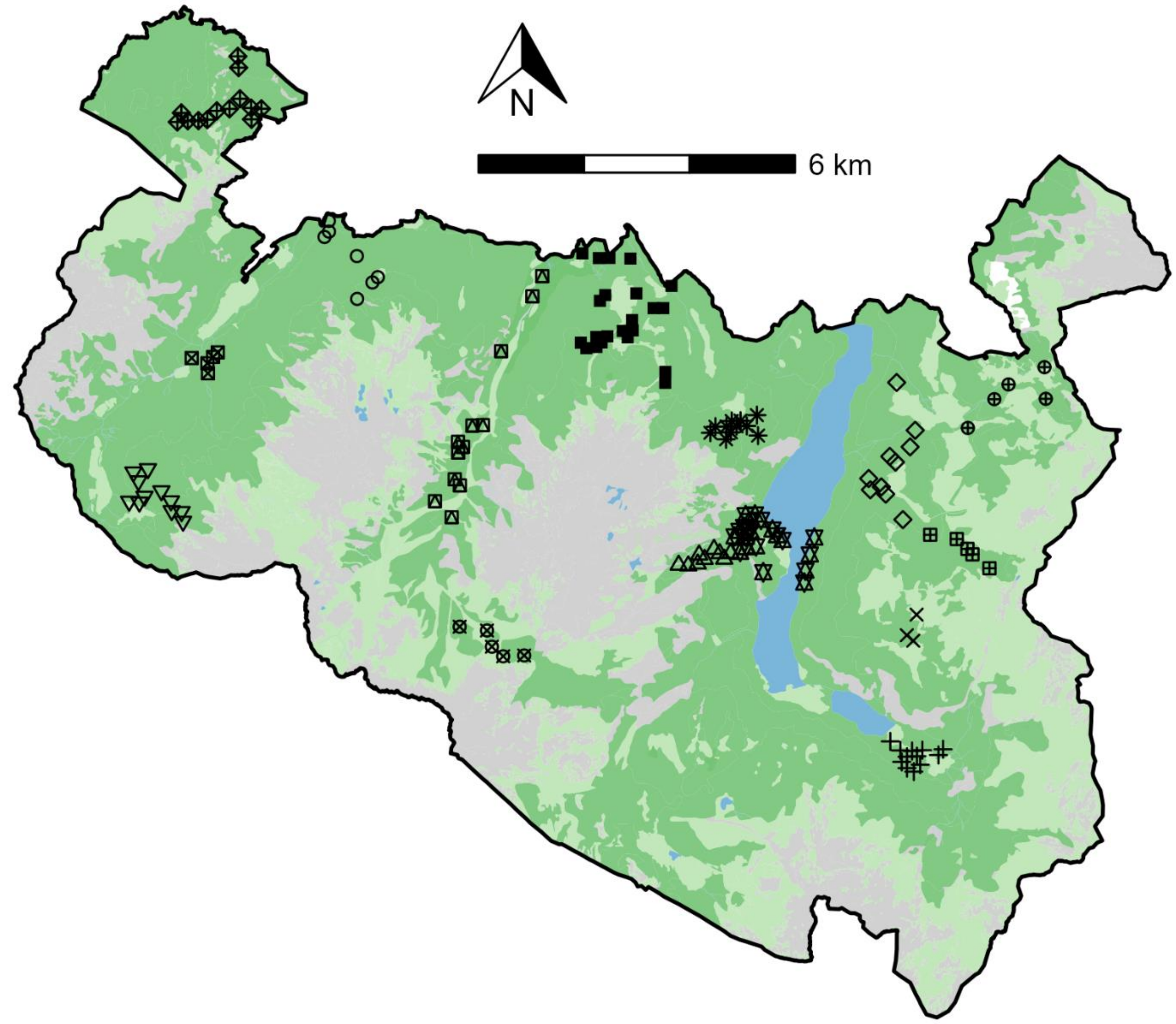

Figure S1 Plot locations at Berchtesgaden National Park. Coordinates were measured using a Trimble r12i GNSS receiver during field sampling. Shapes represent the groups we used as random intercept in our statistical models to account for spatial autocorrelation. Colours represent the main habitats (dark green = forest, light green = open habitats, grey = rock, blue = water/ice) based on data provided by the national park administration.

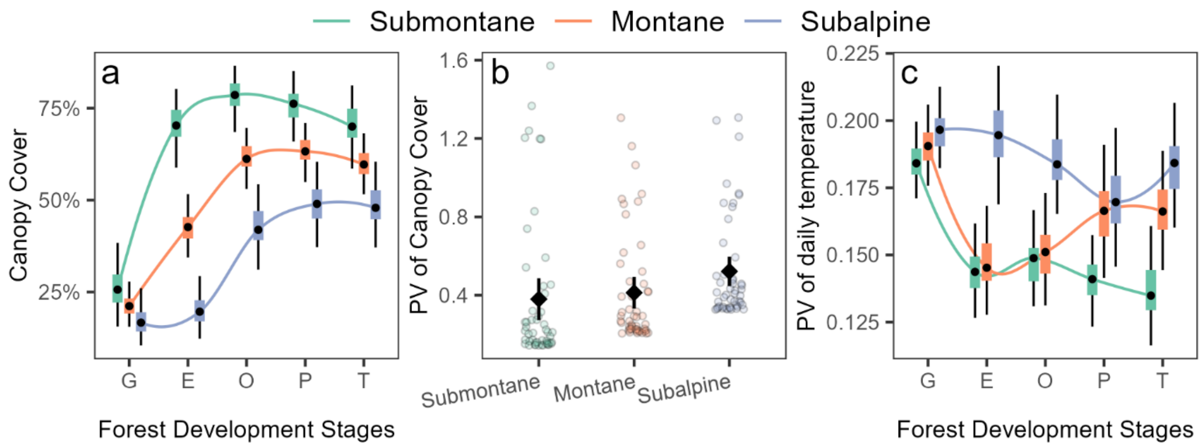

Figure S2 Canopy cover (a & b) was assessed through LiDAR and temperature (c) was measured through TMS-4 TOMST logger at the plot centre. Proportional variability[1] (PV; b & c) was calculated for each plot and day. Canopy cover (a) and PV of daily temperature (c) show predictions from Bayesian multilevel models, summarized by the MAP (Maximum A Posteriori) and 95% and 50% (thick bars in a & c) HDIs (Highest Density Intervals). We fitted a loess curve (a & c) to the MAP for better visualization. Estimates and 95% CIs for the PV of canopy cover (b) was calculated through jackknifing[2]. Points in b show pseudo-values from jackknifing. A detailed description of data acquisition, processing and modelling is provided in the chapters canopy cover and microclimate & statistical analysis below.

Classification of forest developmental stages

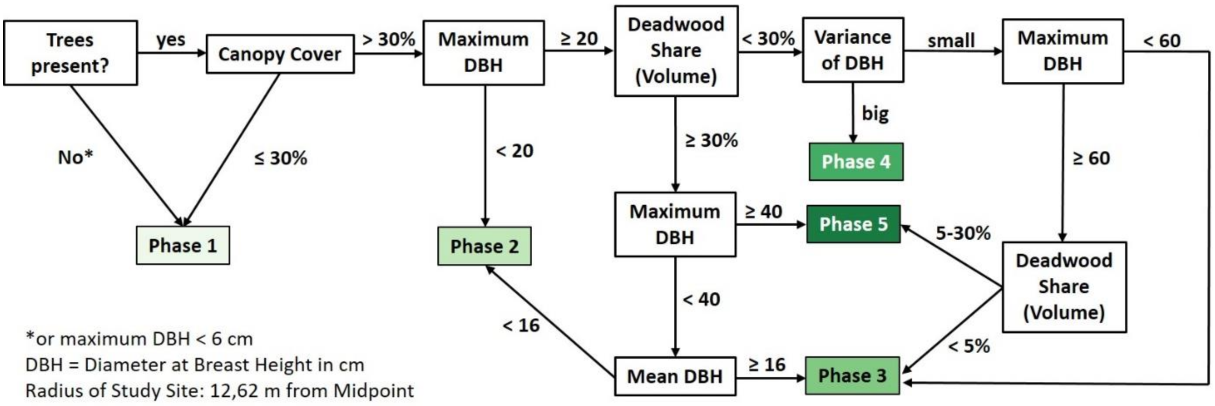

Figure S3 Adjusted classification protocol based on Zenner et al.[3].

Compilation of selected plots

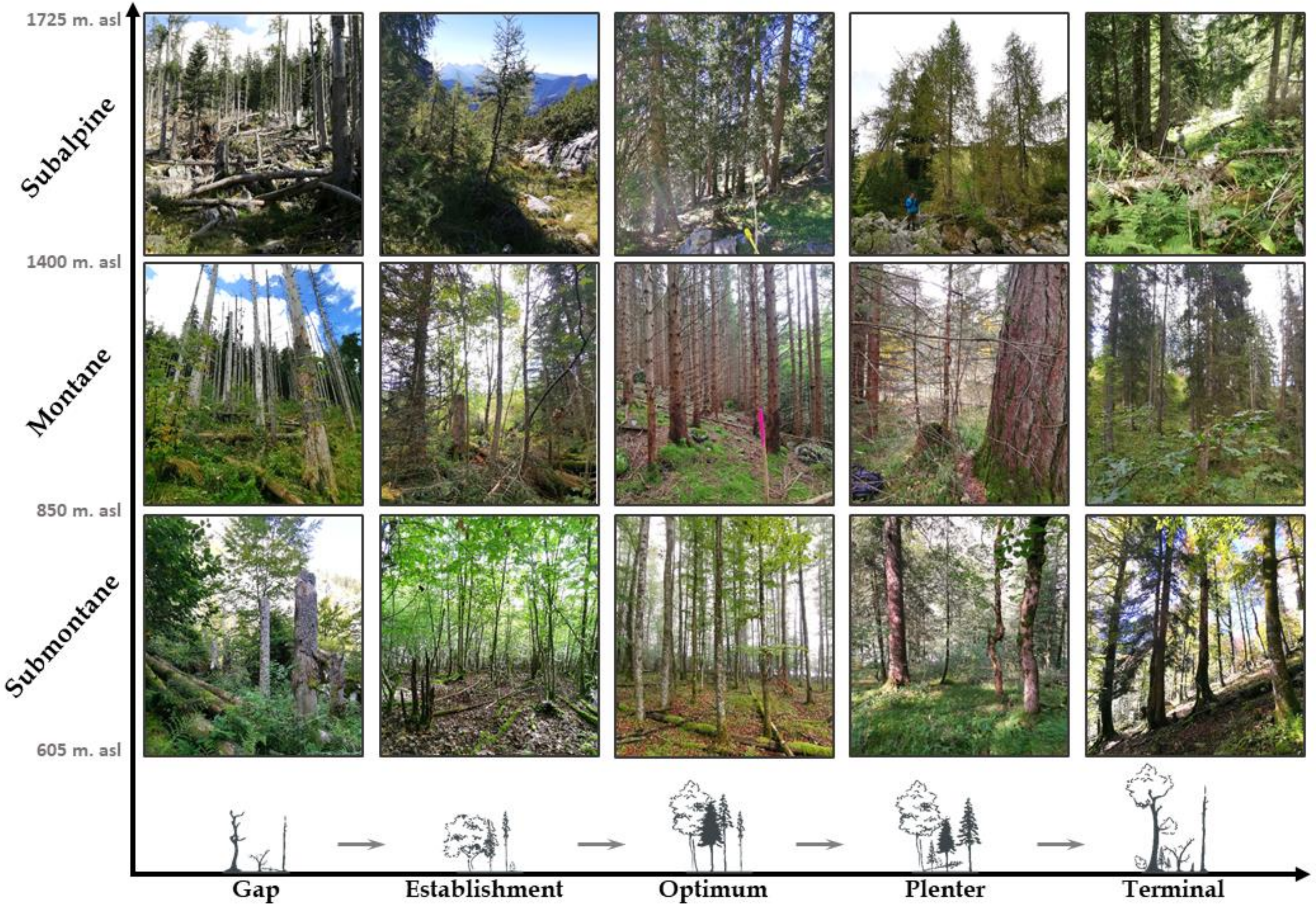

Figure S4 Compilation of the forest developmental stages along the elevational gradient.

Field samplings:

Soil bacteria and fungi: To record bacterial and fungal communities in the soil, we took four soil subsamples at each plot between June 21st and October 25th (Fig. S5). Subsamples were taken at approximately 3 m distance from the plot centre in each of the four cardinal directions. We removed litter (leaf litter, branches, cones), inserted a metal frame (10 cm x 10 cm) into the soil and extracted the organic layer (humus). If the organic layer was less than 10 cm thick, we subsequently used a metal tube (diameter: 5 cm, length: 10 cm) to extract a sample of the mineral layer from underneath. This was the case at most of our study plots. If the organic layer was thicker than 10 cm (tangel humus), we took no subsample of the mineral layer. If the soil consisted mainly of loose rocks and gravel mixed with organic and mineral soil, it was not possible to take a volume-based sample. Instead, we dug a 10 cm deep hole with a vertical wall and extracted an approximately 10 cm x 10 cm x 10 cm subsample. If there was an organic layer on the gravel, we took a 10 cm x 10 cm subsample of the organic layer. We removed all living roots bigger than 2 mm in diameter from the subsamples, pooled and mixed all subsamples per type (i.e., organic, mineral, tangel humus, gravel) and plot. A 40 – 100 g sample was taken per type and study plot, directly cooled in the field and frozen at -20°C on the same day.

Plants: The vegetation was sampled on a 200 m^2 quadratic area around the plot centre separately for the herb (<1 m height) and shrub layer (>1-5 m height) between late May and early August 2021 (Fig. S5).

Arthropods: We used one malaise, three pitfall, and one light trap per plot to collect arthropods. We used this combination of traps as they account for diverging habitats of species groups (i.e., malaise and light traps: flying insects, pitfall: ground and litter dwelling arthropods). We installed the traps after snow melt, starting at the beginning of May in lower elevations and finishing installation in mid-June 2021 at the highest plots. Traps were active until the late growing period (early September) and emptied every two (malaise) and four weeks (pitfall) (Fig. S5). We arranged the pitfall traps in a triangle with a distance of approximately 5 m between each trap and with the malaise trap in the centre. We placed the malaise trap with the collecting bottle pointing approximately southward while avoiding that the entrance to the trap was blocked by high vegetation. The Malaise traps resembled a Townes Malaise trap but with a black roof and slightly smaller in size (height front: 0.90 m; height rear: 0.60 m; length: 1.60 m). Collecting bottles were filled with 70% ethanol and stored under dark and cool conditions until shipping to the laboratory for DNA metabarcoding (see below). Pitfall traps consisted of 400 ml plastic cups (diameter: 8.5 cm) filled with copper-sulfate and a transparent plastic roof to protect them from rain and litter. Pitfall trap samples were transferred into 70% ethanol. Of the three pitfall trap samples per plot and month, we randomly selected two in order maintain comparability across plots per census as single pitfall traps were occasionally destroyed. We separated five taxonomic groups in the laboratory (woodlice, millipedes, centipedes, ants, and beetles), which were subsequently identified to species level by respective experts (see acknowledgements). While beetles were identified for the entire sampling period, the other taxa were only identified for the census in August (Fig. S5). In total, we collected 1,098 malaise trap samples, 985 pitfall trap samples with beetles, 256 with ants, 252 with millipedes, 216 with woodlice, and 211 with centipedes. We used light traps (white vane traps[4] in combination with LepiLED mini lamps[5] (Insects & Lights, Jena, Germany)) to collect moths during one night per plot between mid-June and late July 2022 using chloroform as killing agent (Fig. S5). Samplings were conducted during nights without rain and strong wind. Traps were installed between two trees near the plot centre and LED lamps were active from one hour before sunset (approx. 8:00 pm) until one hour after sunrise (approx. 6:30 am). Moths were transferred into a small plastic box and stored in a freezer until they were identified to species level by an expert (see acknowledgements).

Birds: We used bioacoustic audio recorders (BAR, Frontier Labs, Salisbury, Australia) to record birds between late March and mid-August 2021 during four recording events covering one morning each (Fig. S5). Eleven subalpine plots, however, could not be accessed in March due to the risk of avalanches and thus one sampling is missing. Depending on the availability of recorders and work capacity, we covered either all or approximately half of the plots of a chosen area (Fig. S1), covering all three elevation zones from the beginning on (Fig. S5). After one night, we collected the data and moved the recorders to the remaining plots in the area or to the next area. We recorded only on days with no or negligible rain and low wind speed. Recorders were attached to a tree as close as possible to the plot centre at a height of approximately 1.80 m. We recorded from one hour before to four hours after sunrise every twelve minutes for a duration of two minutes. In addition, we included recordings from two hours before sunrise during the first sampling event to increase the chance of recording owls, which typically sing early in the season and day. For species identification, we selected the first two minutes of every hour starting from one hour before sunrise. In case of noise (e.g., running water, cowbells, vehicles) we selected another recording as close in time as possible to the original one. In total, we had 127 plots with 21 recordings (i.e., 42 minutes) for species identification. Due to noise or failure of the recorder, we had one plot with 20, 19 plots with 16, two plots with eleven, and one plot with nine recordings. Ornithologists (see acknowledgments) identified vocalising species and documented as presence/absence for each recording. Recorder settings: Gain 40 dB, rate = 44.1 kHz.

Bats: We recorded Bats using ultrasonic recorders (batcorder 3.1, ecoObs, Nuremberg, Germany) for two nights. The first survey was conducted between June 3rd and August 3rd 2021, and the second between July 10th and September 23rd 2021 (Fig. S5). Depending on the availability of recorders and work capacity, we covered either all or approximately half of the plots of a chosen area (Fig. S1). After one night, we collected the data and moved the recorders to the remaining plots in the area or to the next area. Recording was restricted to nights without rain and with low wind speed. We attached one recorder to a tree at the outer border of the plot at a height of approximately 2 m and without any vegetation within approximately 3 m in front of the microphone. The recorders were tilted upwards (20-45°) and the microphone pointed approximately towards the plot centre to sample the species present at the plot. Recording started one hour before sunset (approx. 8:00 pm) and ended one hour after sunrise (approx. 6:30 am). Batcorder settings: Quality: 20, Threshold: -27dB, Posttrigger: 400 ms, Critical frequency: 16 kHz, Noise filter: off. Recordings were first analysed using batIdent, a software for automated species identification (ecoObs, Nuremberg, Germany). BatIdent assigns one or more species or species groups to each recording. Initial checking of random sequences confirmed high accuracy of the algorithm for the identification of *Pipistrellus pipistrellus* and the Myotis group that includes *Myotis brandtii*/*mystacinus*. Sequences containing other species or groups were re-checked manually by comparing call structure, especially start-, end- and peak frequencies, to identification keys[6–9] using the bcAnalyze 3 software identification (ecoObs, Nuremberg, Germany) and corrected if necessary.

Large mammals: We used wildlife cameras (Cuddeback G Power House Black Flash 20MP, Green Bay, United States) to record mammals for two periods with a duration of approximately 14 days each. The first sampling took place between May 4th and September 8th 2021, the second sampling between July 8th and October 21st 2021 (Fig. S5). Depending on the availability of cameras and work capacity, we covered either all or approximately half of the plots of a chosen area (Fig. S1), covering all three elevation zones from the beginning on (Fig. S5). After the 14 days, we collected the data and moved the cameras to the remaining plots in the area or to the next area. We installed the cameras on trees or wooden posts at a height of 0.5 m south of the plot centre and facing to the north, with a minimum sight distance of 8 m. Images were checked individually and species were identified. In total, we had 124 plots with 28 recording days. Due to technical problems, we had 19 plots with 27, one plot with 26, and six plots with 14 recording days. Camera setting: Mode: ADV Mode, D/Delay: FAP (fast as

possible), D/Images: 3 images, D/Video: off, D/Lapse: 24 Hrs, N/Delay: FAP, N/Images: 3 images, N/Video: off, Aspect: full, Zone: wide, IR Mode: far, Image SZ: 5 MP, Lapse SZ: 1 MP, DST Mode: off.

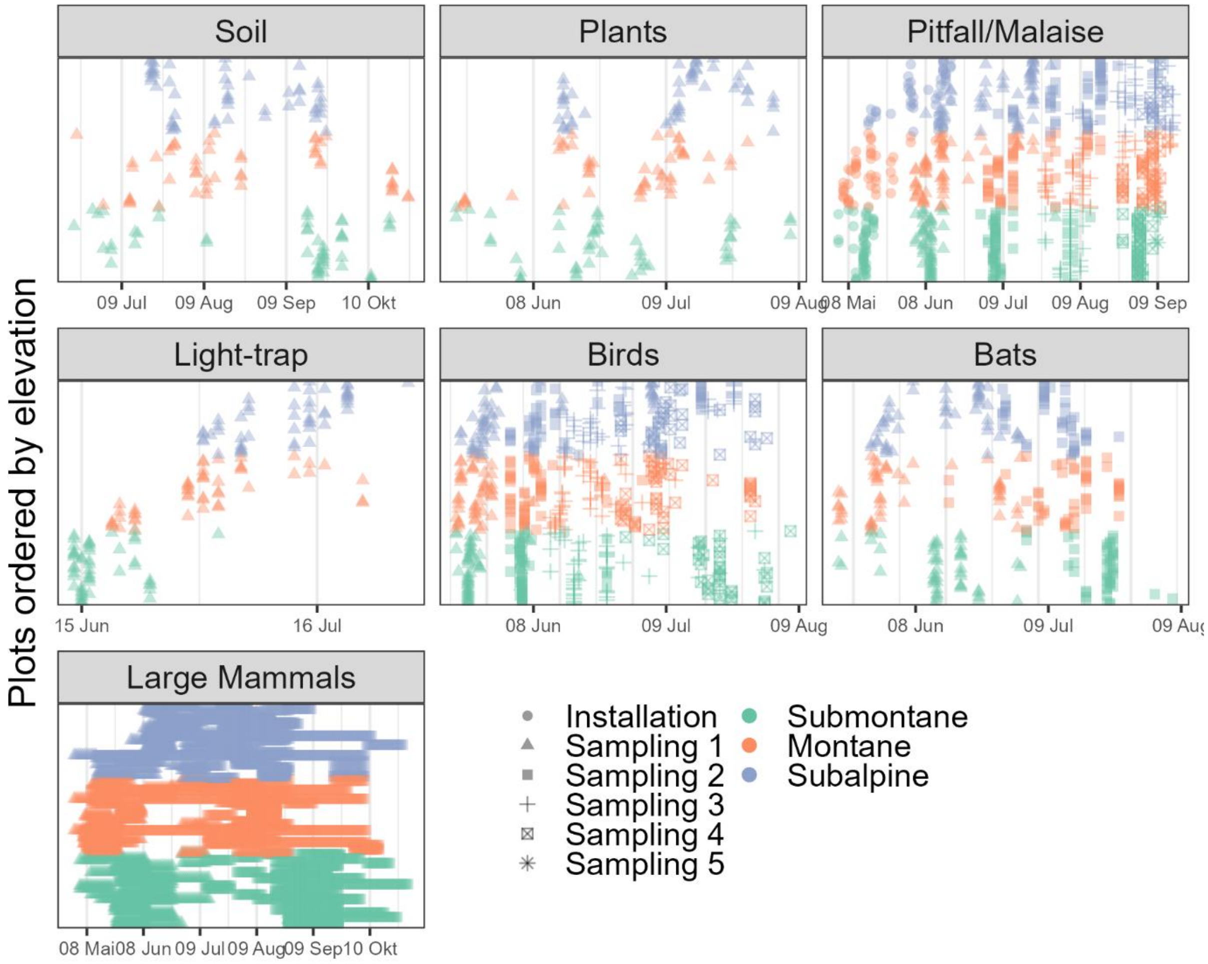

Figure S5 Sampling dates for each sampling method. Note, installation and last sampling of pitfall and malaise traps were the same, but malaise trap samples were collected in intervals of two weeks, which is not shown for clearer visualization.

DNA Metabarcoding

Arthropods: To reduce the total number of samples, we pooled two subsequent Malaise trap samples in chronological order. If the number of samples had an odd number, we omitted the first or last sample from pooling in such a way that the pooled samples best fit the months. On a subset of eight plots (intensive monitoring plots), we did not pool the samples from June and July, but followed the same procedure when having an odd number of samples of the remaining months. After pooling, we had 587 samples for DNA metabarcoding that were first sorted in two fractions (smaller or larger 6.5 mm) using cascade sieves. Fractions were dried overnight at room temperature to remove the resdiual ethanol. Dried samples were suspended in a tissue-lysis buffer (Tris-HCl pH 7.5 200 mmol/L, NaCl 250 mmol/L, EDTA 25 mmol/L, SDS 0.5 %) and then homogenized with stainless steel beads using an ULTRA-TURRAX® Tube Drive (IKA-Werke GmbH & CO. KG, Staufen, Germany). Equal amounts of homogenates from each fraction were mixed to a composite sample.

Arthropod DNA was isolated from a 200 µL sample of the composite samples using the NucleoSpin® tissue kit (Macherey-Nagel GmbH & CO. KG, Düren, Germany). DNA extracts were used as templates for the amplification of a 313 bp fragment of the cytochrome c oxidase I (COI) using modified mlCOIintF/jgHCO2198 primers[10,11].

Dual indexes and Illumina sequencing adapters were attached to the amplicons using the Nextera XT Index Kit (Illumina Inc., San Diego, CA, USA) and the KAPA HiFi HotStart ReadyMix (Roche Diagnostics Deutschland GmbH, Mannheim, Germany) following the manufacturer's protocol. Libraries were purified using the NucleoMag NGS Clean-up, quantified using the Promega Quantifluor ONE dsDNA system (Promega, Mannheim, Germany), and pooled aequimolarily. Pooled libraries were sequenced on an Illumina MiSeq system using v3 chemistry (2 x 300 bp) by biome-id (Wilhelmshaven, Germany).

Paired-end reads were assembled using vsearch[12] (v. 2.18.0) setting a minimum overlap of 100 bases. Assembled reads were then demultiplexed according to the sample-specific dual indexes and PCR primers were removed using cutadapt[13]. All contigs were filtered for uncalled bases ("N") and dereplicated with vsearch. Dereplicated sequences were clustered in Operational Taxonomic Units (OTUs) using a 97 % similarity threshold. Chimera sequences were detected and removed using the UCHIME algorithm[14] and an OTU-table was constructed using the usearch_global algorithm[15]. All dereplicated sequences were used as query file and the list of OTUs as the database. All non-chimeric OTUs composed of more than one sequence (non-singletons) were then taxonomically assigned using the blastn algorithm[16] on a custom reference database of European terrestrial arthropods, which was assembled from BOLD and NCBI public data and limited to records from Germany and the adjacent countries (status: July 2022). We used the Barcode Index Number (BIN) system of BOLD (Barcode of Life Datasystems) to maintain the same level of identification for all taxonomic groups, since OTUs tend to overestimate richness in some orders such as orthopterans or syrphidae. The utility of BINs in assessing biodiversity has been largely demonstrated[17–19].

Soil fungi and bacteria: Soil samples were sieved (mesh size: 250 µm) and the organic and mineral samples were pooled into a composite sample of 10 ml. The mixing ratio organic:mineral was always 1:1 (i.e. 5 ml each), but within these categories we pooled samples according to the availability of samples per type. This means, subsamples from a plot with, for example, three humus, one tangel humus, and four mineral subsamples were mixed with a 3:1:4 ratio (i.e. 3.75 ml humus, 1.25 ml tangel humus, 5 ml mineral) to keep the organic vs. mineral ratio constant but consider the humus vs. tangel humus ratio. If only an organic or mineral sample was available, we took a 10 ml sample to keep the volume constant (2:0 / 0:2 mixing ratio). For further examples of mixing ratios, see Table S1.

Table S1 Examples of different mixing ratios of the soil samples for bacteria and fungi barcoding.

Subsample	Organic		Mineral	
	Humus	*Tangel*	*Mineral*	*Gravel*
1	x		x	
2	x		x	
3	x		x	
4			x	
**Ratio**	1	0	1	0
**Volume [ml]**	5	0	5	0

Subsample	Organic		Mineral	
	Humus	*Tangel*	*Mineral*	*Gravel*
1		x		
2		x		
3		x		
4		x		
**Ratio**	0	2	0	0
**Volume [ml]**	0	10	0	0

Subsample	Organic		Mineral	
	Humus	*Tangel*	*Mineral*	*Gravel*
1	x	x	x	
2	x	x	x	
3				
4				
**Ratio**	1	1	2	0
**Volume [ml]**	2.5	2.5	5	0

Subsample	Organic		Mineral	
	Humus	*Tangel*	*Mineral*	*Gravel*
1	x	x	x	x
2		x		x
3				
4				
**Ratio**	1	2	1	2
**Volume [ml]**	1.67	3.33	1.67	3.33

Subsample	Organic		Mineral	
	Humus	*Tangel*	*Mineral*	*Gravel*
1	x	x	x	
2	x		x	
3			x	
4				
**Ratio**	2	1	3	0
**Volume [ml]**	3.33	1.67	5	0

Subsample	Organic		Mineral	
	Humus	*Tangel*	*Mineral*	*Gravel*
1	x	x	x	
2		x		
3		x		
4				
**Ratio**	1	3	4	0
**Volume [ml]**	1.25	3.75	5	0

Fungi and bacteria DNA was isolated from a 250 mg sample of the composite soil samples using the DNeasy PowerSoil Pro Kit (Quiagen GmbH, Hilden, Germany). DNA concentrations were quantified using a NanoDrop UV-Vis spectrophotometer (Peqlab Biotechnologie GmbH, Erlangen, Germany). Both PCR reactions were performed in 25 µL triplicate reactions containing 12.5 µL of GoTaq Green

Mastermix (Promega, Madison, WI, USA), 10 µM of each primer (fungi: ITS4/fITS7, bacteria: 515F/806R), and 1–2 µL template DNA, following the protocols described[20,21].

Indexing and sequencing was done similarly to the arthropod samples, carried out by AIM (Leipzig, Germany), but a Fluoroskan plate reader (Thermofisher) instead a Promega Quantifluor ONE dsDNA system (Promega, Mannheim) was used for quantification.

Paired-end reads were assembled using usearch (v. 11.0.667). PCR primers were removed using cutadapt (fungi: v3.5 with Python 3.10.6; bacteria: v4.1 with Python 3.9.12). Quality filtering and dereplication was done by using vsearch[12] (v. 2.21.1). The minimum length of a sequence was kept above 300 for fungi and above 200 for bacteria with a maximum of one expected error for both taxa. All singletons were discarded in the same step. Dereplicated sequences were clustered in Operational Taxonomic Units (OTUs) using a 97 (fungi) and 98 % (bacteria) similarity threshold. Chimera sequences were detected and removed using the UCHIME algorithm[14] as implemented in vsearch. All non-chimeric OTUs were taxonomically assigned using the blastn algorithm[16] against the BOLD reference database (fITS2) and the NCBI public data (fITS2, 16S). We applied a sample-based filtering step discarding all OTUs with less than 0.01% of the total reads per sample and used the resulting number of OTUs as a proxy for bacteria and fungi richness.

Data preparation

We excluded Cyanobacteria, Chloroplasts, and plants in the barcoding data from bacteria or fungi. We also excluded species that are not in focus of the study, such as tree (but not shrub/tree) species according to life form data compiled from the TRY plant trait database[22,23], as well as water associated insects from malaise samples (Trichoptera, Plecoptera, Ephemeroptera, Odonata) and bird species (*Anas platyrhynchos*, *Aythya fuligula*, *Bucephala clangula*, *Fulica atra*). From the Malaise trap data, we only used data from the class of insects, as these made up about 96% of all arthropod BINs. We pooled the data from pitfall and light traps (i.e., woodlice, millipedes, centipedes, ants, beetles, moths), forming the arthropods$_{TAX}$ group, as well as the data from birds, bats, small and large mammals, forming the vertebrates group. The malaise trap data consisted of 16 insect orders (Archaeognatha, Blattodea, Coleoptera, Dermaptera, Diptera, Hemiptera, Hymenoptera, Lepidoptera, Mecoptera, Megaloptera, Neuroptera, Orthoptera, Psocodea, Raphidioptera, Siphonaptera, Thysanoptera). We finally pooled the data from multiple samplings at plot level.

Canopy cover and microclimate

We used LiDAR data from 2021[24,25] to quantify canopy cover at plot level ($r = 12.62$ m) as the proportion of returns above 5 m using the lidR package[26]. To quantify microclimate, we used Tomst TMS-4 loggers[27] to record temperature every 15 minutes at -6, 2 and 15 cm at the plot centre. We calculated the Proportional Variability (PV)[1] to quantify the variability in canopy cover and microclimate. Instead of measuring the deviation from an average as known from the standard deviation (SD) or coefficient of variation (CV), PV quantifies variability by simply comparing all numbers to each other. PV is a truly proportional first order measure of variability for Gaussian and non-Gaussian data and robust against rare events[28]. For canopy cover, we calculated the PV across plots of each elevation belt and estimated the mean and 95% confidence interval for each elevation belt through jackknifing[2]. For microclimate, we used data from 2 cm above ground as data from 2 and 15 cm above ground were highly correlated ($r = 0.97$, $p < 0.001$). We calculated the PV for each plot and day during pitfall/malaise trap activity period and modelled the PV as described below in the statistical methods.

Statistical analysis

Canopy cover and microclimate

To assess how canopy cover and micro-climatic variability change along the developmental and elevational gradient, we fitted multilevel models within a Bayesian framework using the brms package[29]. For canopy cover we used an interaction term between the forest developmental stage (categorical) and elevation (continuous, z-transformed) as predictors. We fitted the models using a Beta probability distribution and added a variable that groups study plots in close proximity as random intercept to account for spatial autocorrelation[30] (groups are shown in supplementary Fig. S1). For the PV of microclimate, we likewise used an interaction term between the forest development stage and elevation as predictors. Because PV followed an inverse sigmoid (increasing-horizontal-increasing) pattern with increasing elevation, we added elevation as fourth order polynomial using the poly function to adequately capture this trend. To account for (daily) repeated measures, we added the plot id as random intercept. We used a Beta probability distribution and added elevation and day of year as z-score.

Model specifications

We ran models for specialization, species richness, canopy cover, and PV of microclimate using four chains, each with 4,000 iterations including 2,000 iterations as warm-up. For beta diversity models, we had to increase to 5,000 iterations in order to ensure that the rank-normalized effective sample size of the bulk of the distribution exceeded 400 for each model parameter[31]. We specified weakly informative priors (N(0, 10) for categorical and N(0, 2) for z-scores) to restrict the parameters to plausible ranges and to stabilise computations while only moderately regulating the model[32,33].

Model evaluations

We checked model convergence by means of trace plots, autocorrelation, $\hat{R}$-values, and rank-normalized bulk effective sample size[31]. The latter two are shown in the model summaries below (Tables S4, S7a-S7c, S9, S11, and S12). We used the DHARMa package to check residuals for normality and heteroscedasticity, as well as for spatial autocorrelation of all except beta diversity models[34]. Checks for spatial autocorrelation showed that adding a variable that groups study plots in close proximity as random intercept strongly reduced the spatial autocorrelation and often improved the residual patterns. We further checked multicollinearity (VIF) and evaluated goodness-of-fit by means of posterior predictive checks using the Bayesplot package[35] and performance indices using the performance package[36]. Performance indices are provided in Table S2. Traceplots, MCMC autocorrelation figures, and results of the posterior predictive, residual, and multicollinearity checks are not provided in the manuscript nor the supplemental material, but can be reproduced using the code published with this manuscript.

Table S2 Goodness-of-fit of each fitted model.

Model	Taxon	R2	R2_adjusted	RMSE	R2_marginal
Specialization	Bacteria	0.41	0.39	0.26	
	Fungi	0.76	0.69	0.14	
	Plants	0.55	0.48	0.21	
	Arthropods[TAX]	0.64	0.62	0.19	
	Insects[BIN]	0.76	0.70	0.14	
	Vertebrates	0.57	0.49	0.20	
Species richness	Bacteria	0.15		264.36	0.09
	Fungi	0.27		123.26	0.18
	Plants	0.36		11.13	0.26
	Arthropods[TAX]	0.58		15.86	0.49
	Insects[BIN]	0.59		175.59	0.46
	Vertebrates	0.31		3.89	0.13
Jaccard dissimilarity	Bacteria	0.60		0.04	0.10
	Fungi	0.68		0.02	0.09
	Plants	0.60		0.04	0.10
	Arthropods[TAX]	0.74		0.03	0.45
	Insects[BIN]	0.76		0.02	0.25
	Vertebrates	0.59		0.05	0.19
Canopy cover		0.65		0.15	0.58
Microclimate		0.30		0.09	0.19

Concept Difference of Differences

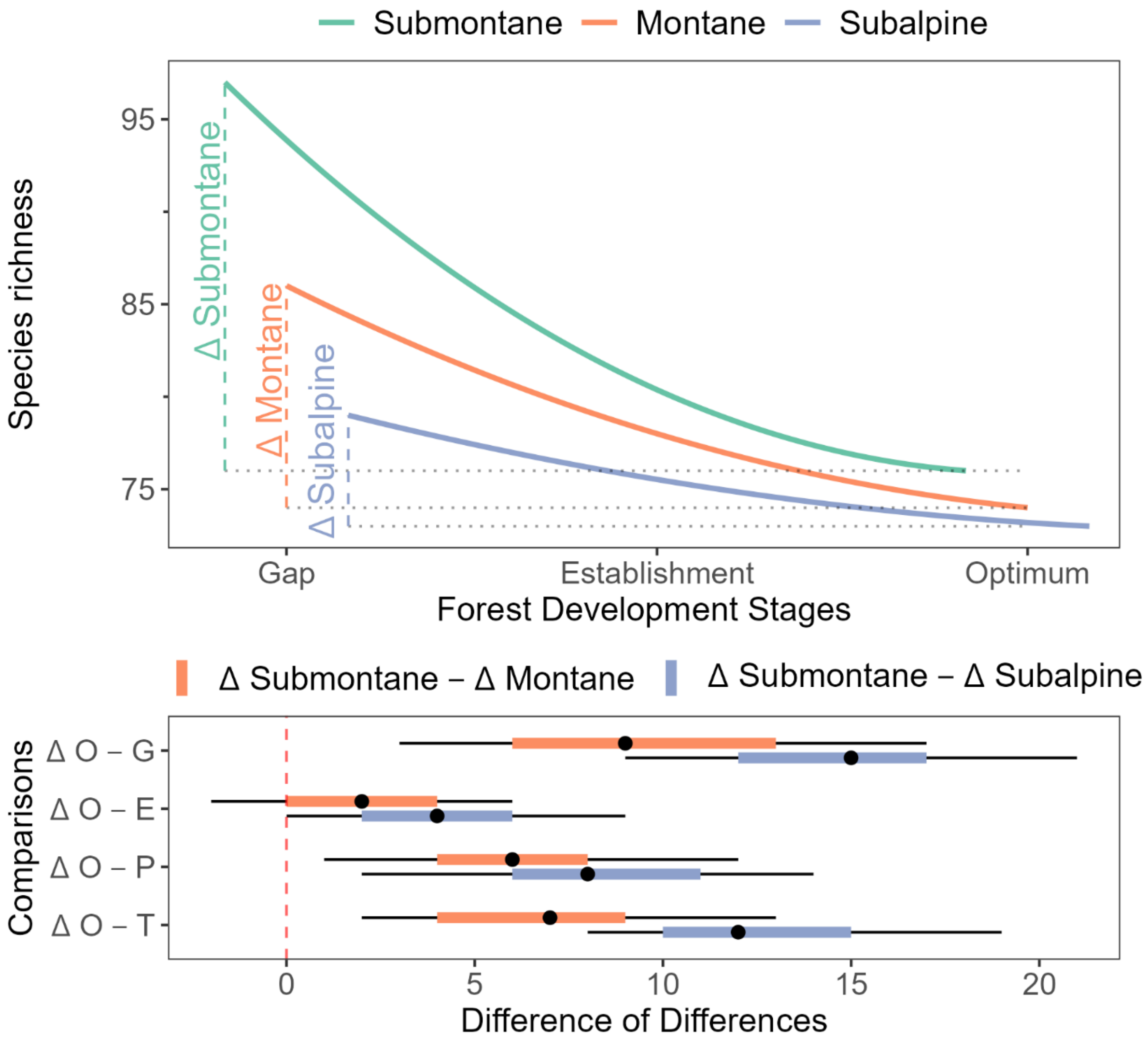

Figure S6 Computation of the difference of differences. The top figure shows species richness for the three elevation belts along half of the forest developmental gradient. We first computed the absolute difference between the optimum and each other developmental stage, separately for each elevation belt (exemplary shown for ΔO-G). We then subtracted the absolute differences of each optimum-[developmental stage]-comparison (i.e. Δ O - G, Δ O - E, Δ O - P, Δ O - T) of the montane and subalpine zone from the absolute difference of the corresponding optimum-[developmental stage]-comparison of the submontane belt. Our hypothesis is supported by both positive values and larger differences in the submontane-subalpine compared to the submontane-montane comparison, as shown in the bottom figure. Results are shown in Table S8 and Fig. S10.

Results

Descriptive summary

Table S3 Descriptive summary of the results. Habitat diversity was calculated separately for each elevational zone using the reciprocal of the Simpson index based on the proportional use of developmental stages. For calculating the SES we simulated 500 random communities, subtracted the average from the observed habitat diversity and divided by the standard deviation, and finally multiplied by -1 to receive a measure for habitat specialization. Species richness represents the number of species/BINs/OTUs found at a plot, Jaccard dissimilarity was calculated between each plot pair of a different developmental stage separately for each elevational zone, and the last column shows the total number of species/BINs/OTUs found in this study.

Taxon	Habitat diversity		SES of specialization		Species richness		Jaccard dissimilarity		No. species/ BINs/OTUs
	avg.	range	avg.	range	avg.	range	avg.	range	total
Bacteria	4.2	(3.4-4.5)	0.07	(-0.32-1.54)	1,382	(789-2642)	0.68	(0.48-0.9)	12,687
Fungi	3.6	(3.1-4.2)	-0.11	(-0.64-1.32)	548	(136-839)	0.84	(0.68-0.95)	9,173
Plants	4.1	(3-4.6)	-0.22	(-0.87-1.26)	52	(21-87)	0.68	(0.48-0.9)	443
Arthropods_{TAX}	4.1	(3.7-4.5)	-0.22	(-1.04-0.71)	103	(40-163)	0.73	(0.5-0.91)	958
Insects_{BIN}	4.1	(3.6-4.4)	-0.06	(-0.78-0.83)	1,035	(381-1766)	0.73	(0.51-0.89)	8,335
Vertebrates	4.5	(4-4.8)	0.15	(-0.52-1.13)	28	(17-39)	0.54	(0.15-0.8)	105

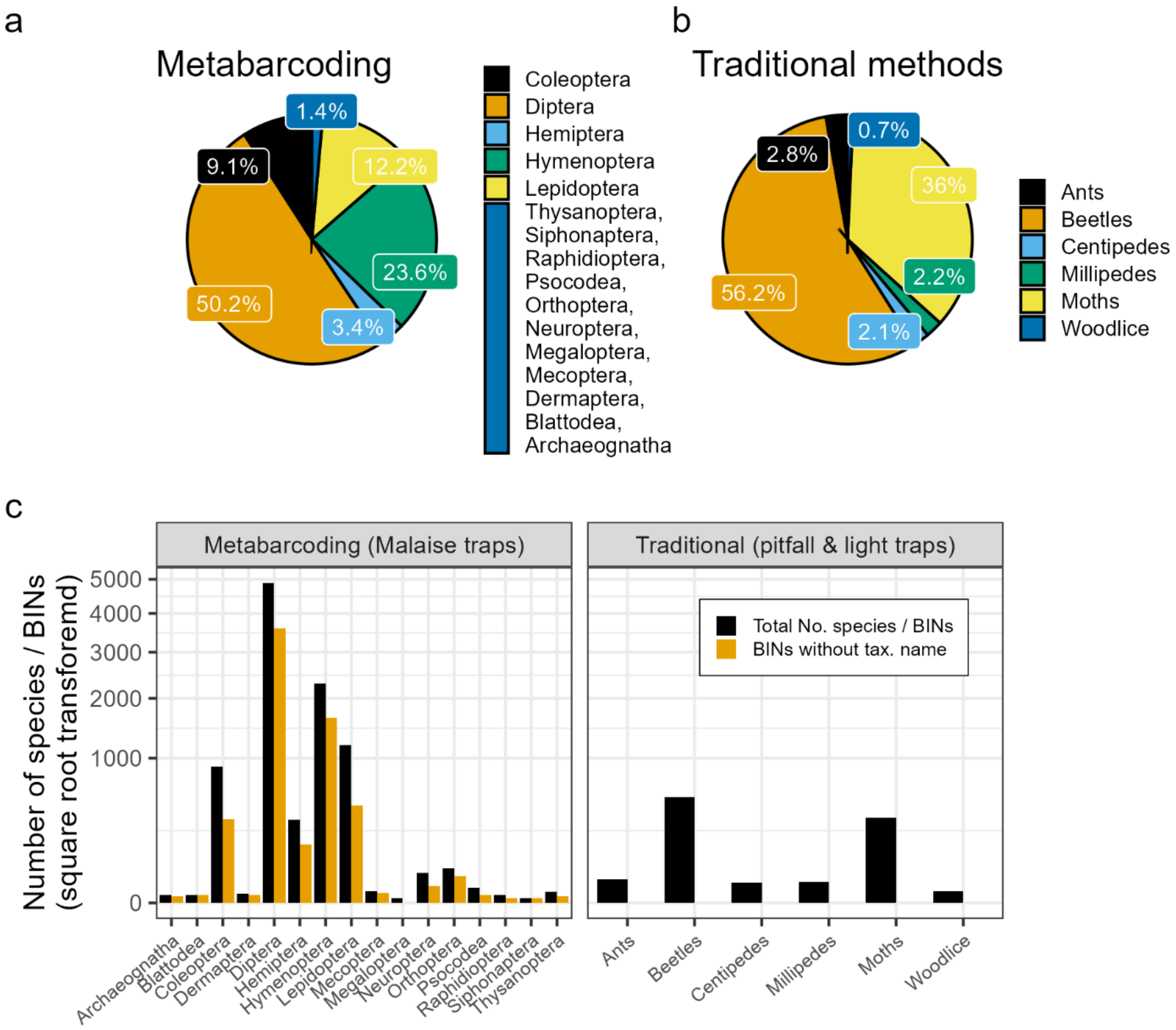

Fig. S7 shows the distribution of BINs/species (a & b) and the absolute number of BINs/species including BINs without a taxonomic name (c) of the analysed taxa for species identification through metabarcoding and traditional expert annotations.

Specialization

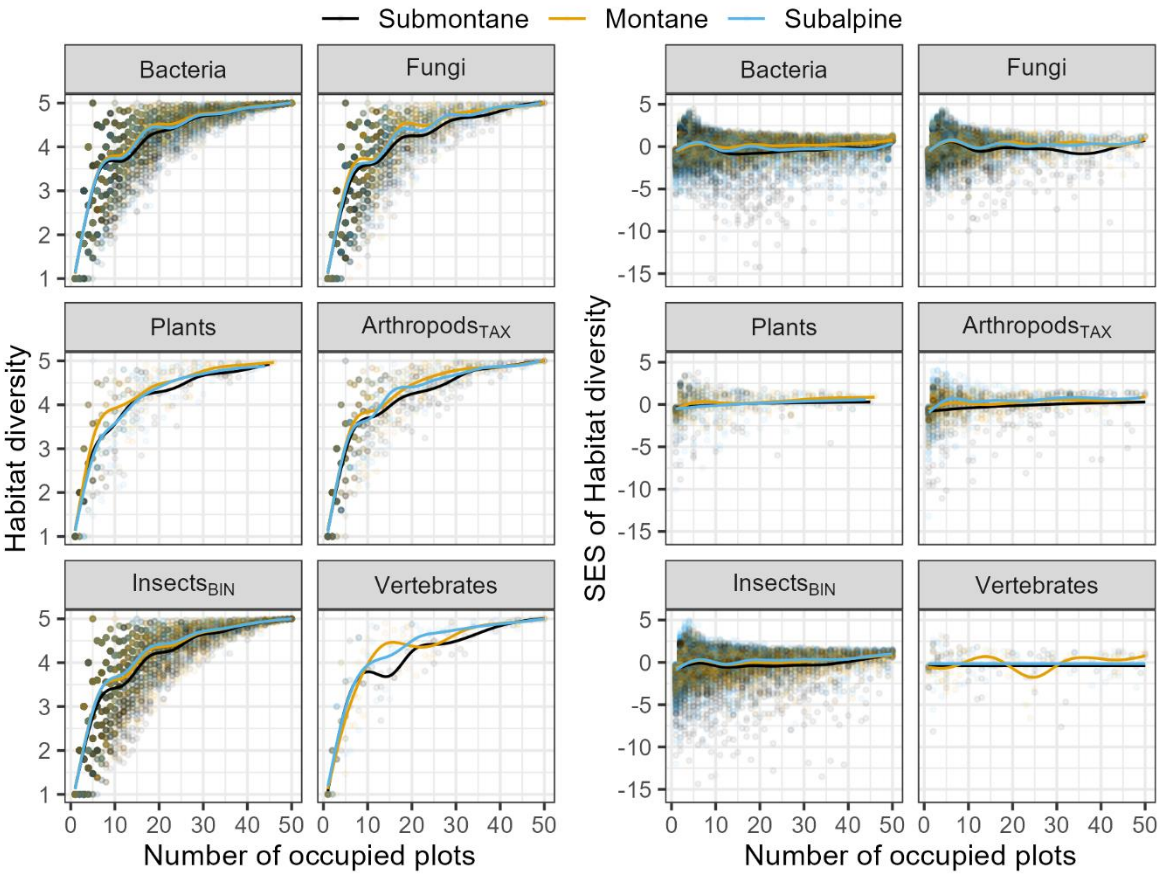

Fig. S8 Habitat diversity increases with an increasing number of occupied plots and using the Standardized Effect Size (SES) from the null model allows to account for that.

Table S4 shows the total number of species per taxon and elevational zone, along with the number and proportion of species lost due to low variation across simulated communities and thus a standard deviation of zero, when computing the Standardized Effect Size of habitat specialization.

taxon	total	present	lost	prop_lost	elev zone
Bacteria	9,251	7,503	1,748	0.189	Submontane
	7,289	6,557	732	0.100	Montane
	8,887	7,242	1,645	0.185	Subalpine
Fungi	6,026	4,977	1,049	0.174	Submontane
	5,509	4,815	694	0.126	Montane
	5,488	4,562	926	0.169	Subalpine
Plants	277	245	32	0.116	Submontane
	284	269	15	0.053	Montane
	307	269	38	0.124	Subalpine
Arthropods[TAX]	644	541	103	0.160	Submontane
	649	570	79	0.122	Montane
	568	489	79	0.139	Subalpine
Insects[BIN]	6,360	5,622	738	0.116	Submontane
	6,213	5,680	533	0.086	Montane
	5,220	4,820	400	0.077	Subalpine
Vertebrates	82	79	3	0.037	Submontane
	85	83	2	0.024	Montane
	85	81	4	0.047	Subalpine

Table S5 Model summaries explaining the SES of specialization as a function of elevational zones for each taxon. MAP = Maximum A Posteriori of posterior distribution, MAP_CI95 = 95% highest density intervals (HDI) of posterior distribution, pd = probability of direction, %inROPE = percentage of the 95% HDI laying within the Region Of Practical Equivalence, ROPE = Region Of Practical Equivalence, Rhat = potential scale reduction factor (should be close to 1 to indicate model convergence), ESS = rank-normalized effective sample size of the bulk of the distribution.

Taxon	Parameter	MAP	MAP_CI95	pd	%inROPE	ROPE	Rhat	ESS
Bacteria	b_Intercept	0.33	[0.2,0.48]	1.00	0	+/-0.04	1	4,233
	b_sigma_Intercept	-0.89	[-1.1,-0.69]	1.00	0	+/-0.04	1	8,643
	b_elev_classMontane	-0.54	[-0.69,-0.4]	1.00	0	+/-0.04	1	4,534
	b_elev_classSubalpine	-0.22	[-0.39,-0.07]	1.00	0	+/-0.04	1	4,057
	b_sigma_elev_classMontane	-1.96	[-2.26,-1.64]	1.00	0	+/-0.04	1	8,912
	b_sigma_elev_classSubalpine	-0.58	[-0.88,-0.29]	1.00	0	+/-0.04	1	9,523
Fungi	b_Intercept	0.43	[0.26,0.6]	1.00	0	+/-0.03	1	4,652
	b_sigma_Intercept	-1.67	[-1.87,-1.37]	1.00	0	+/-0.03	1	5,230
	b_elev_classMontane	-0.79	[-0.97,-0.62]	1.00	0	+/-0.03	1	4,108
	b_elev_classSubalpine	-0.67	[-0.86,-0.5]	1.00	0	+/-0.03	1	4,357
	b_sigma_elev_classMontane	-0.31	[-0.66,0.07]	0.94	5	+/-0.03	1	6,275
	b_sigma_elev_classSubalpine	-0.47	[-0.83,-0.15]	1.00	0	+/-0.03	1	6,564
Plants	b_Intercept	0.18	[-0.01,0.41]	0.96	5	+/-0.04	1	5,327
	b_sigma_Intercept	-1.32	[-1.53,-1.1]	1.00	0	+/-0.04	1	7,213
	b_elev_classMontane	-0.62	[-0.81,-0.4]	1.00	0	+/-0.04	1	4,853
	b_elev_classSubalpine	-0.26	[-0.48,-0.04]	0.99	0	+/-0.04	1	4,951
	b_sigma_elev_classMontane	-0.88	[-1.2,-0.52]	1.00	0	+/-0.04	1	8,279
	b_sigma_elev_classSubalpine	0.04	[-0.29,0.36]	0.60	17	+/-0.04	1	7,522
$Arthropods_{TAX}$	b_Intercept	0.29	[0.16,0.43]	1.00	0	+/-0.04	1	6,778
	b_sigma_Intercept	-1.86	[-2.06,-1.6]	1.00	0	+/-0.04	1	6,514
	b_elev_classMontane	-0.58	[-0.71,-0.44]	1.00	0	+/-0.04	1	6,007
	b_elev_classSubalpine	-0.77	[-0.92,-0.59]	1.00	0	+/-0.04	1	6,869
	b_sigma_elev_classMontane	-0.66	[-1.01,-0.33]	1.00	0	+/-0.04	1	8,717
	b_sigma_elev_classSubalpine	0.70	[0.35,1.01]	1.00	0	+/-0.04	1	9,023
$Insects_{BIN}$	b_Intercept	0.35	[0.18,0.51]	1.00	0	+/-0.03	1	5,435
	b_sigma_Intercept	-1.67	[-1.86,-1.43]	1.00	0	+/-0.03	1	8,691
	b_elev_classMontane	-0.55	[-0.73,-0.4]	1.00	0	+/-0.03	1	5,182
	b_elev_classSubalpine	-0.56	[-0.74,-0.4]	1.00	0	+/-0.03	1	5,538
	b_sigma_elev_classMontane	-0.22	[-0.54,0.1]	0.91	7	+/-0.03	1	8,645
	b_sigma_elev_classSubalpine	-0.50	[-0.88,-0.17]	1.00	0	+/-0.03	1	5,851
Vertebrates	b_Intercept	0.47	[0.33,0.6]	1.00	0	+/-0.03	1	7,025
	b_sigma_Intercept	-1.52	[-1.72,-1.27]	1.00	0	+/-0.03	1	7,621
	b_elev_classMontane	-0.50	[-0.64,-0.33]	1.00	0	+/-0.03	1	7,279
	b_elev_classSubalpine	-0.46	[-0.62,-0.3]	1.00	0	+/-0.03	1	6,544
	b_sigma_elev_classMontane	0.13	[-0.18,0.46]	0.80	12	+/-0.03	1	9,065
	b_sigma_elev_classSubalpine	-0.38	[-0.7,-0.05]	0.99	0	+/-0.03	1	8,675

Table S6 Calculated differences of the SES of habitat specialization between the elevational zones.

Taxon	Comparison	MAP	MAP_CI95	MAP_CI50	pd	%inROPE	ROPE
Bacteria	Submontane - Montane	0.54	[0.4,0.69]	[0.49,0.59]	1.00	0	+/-0.036
	Submontane - Subalpine	0.22	[0.06,0.39]	[0.16,0.27]	1.00	0	+/-0.036
	Montane - Subalpine	-0.32	[-0.39,-0.25]	[-0.34,-0.29]	1.00	0	+/-0.036
Fungi	Submontane - Montane	0.79	[0.62,0.97]	[0.72,0.84]	1.00	0	+/-0.041
	Submontane - Subalpine	0.67	[0.5,0.86]	[0.61,0.74]	1.00	0	+/-0.041
	Montane - Subalpine	-0.11	[-0.2,-0.02]	[-0.14,-0.08]	0.99	4	+/-0.041
Plants	Submontane - Montane	0.62	[0.41,0.82]	[0.52,0.67]	1.00	0	+/-0.041
	Submontane - Subalpine	0.26	[0.05,0.5]	[0.18,0.34]	0.99	0	+/-0.041
	Montane - Subalpine	-0.34	[-0.49,-0.2]	[-0.39,-0.29]	1.00	0	+/-0.041
$Arthropods_{TAX}$	Submontane - Montane	0.58	[0.44,0.71]	[0.52,0.62]	1.00	0	+/-0.025
	Submontane - Subalpine	0.77	[0.59,0.93]	[0.71,0.82]	1.00	0	+/-0.025
	Montane - Subalpine	0.19	[0.06,0.31]	[0.15,0.23]	1.00	0	+/-0.025
$Insects_{BIN}$	Submontane - Montane	0.55	[0.4,0.73]	[0.5,0.61]	1.00	0	+/-0.027
	Submontane - Subalpine	0.56	[0.4,0.74]	[0.5,0.62]	1.00	0	+/-0.027
	Montane - Subalpine	0.00	[-0.09,0.11]	[-0.03,0.04]	0.55	44	+/-0.027
Vertebrates	Submontane - Montane	0.50	[0.33,0.65]	[0.43,0.54]	1.00	0	+/-0.025
	Submontane - Subalpine	0.46	[0.3,0.61]	[0.41,0.51]	1.00	0	+/-0.025
	Montane - Subalpine	-0.03	[-0.14,0.09]	[-0.07,0.01]	0.69	31	+/-0.025

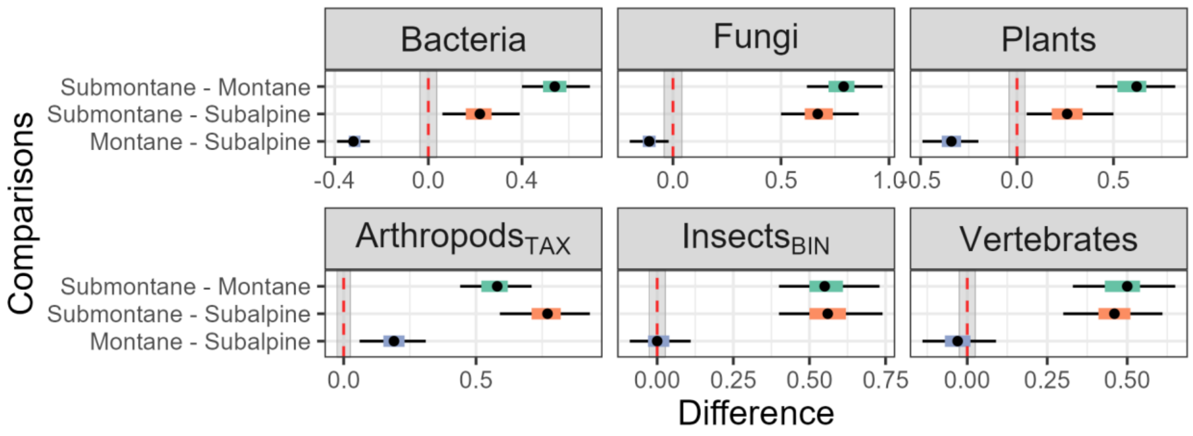

Figure S9 Visualization of the calculated differences of the SES of habitat specialization between the elevational zones shown in Table S5. Points show the MAP and bars the 50% (thick coloured bars) and 95% HDIs. Grey shaded area represents the ROPE.

Species richness

Table S7 Effect of elevation on species richness. MAP was calculated through adding the baseline elevation coefficient (gap stage) to all interaction coefficients of development stage and elevation (Tables S7a-S7c) and average over all. Metrics are explained in table S4. MAP and MAP_CI values are on the log-scale, values in brackets are the back-transformed values using the formula *exp(x)-1* and showing the rate of change.

Taxon	MAP [transf]	MAP_CI95 [transf]	pd	%inROPE	ROPE
Bacteria	-0.007 [-0.007]	-0.053, 0.042 [-0.052,0.043]	0.60	100	+/-0.1
Fungi	-0.014 [-0.014]	-0.077, 0.056 [-0.074,0.058]	0.66	100	+/-0.1
Plants	0.042 [0.043]	-0.014, 0.12 [-0.014,0.128]	0.94	94	+/-0.1
$Arthropods_{TAX}$	-0.176 [-0.161]	-0.228, -0.124 [-0.204,-0.117]	1.00	0	+/-0.1
$Insects_{BIN}$	-0.108 [-0.102]	-0.161, -0.042 [-0.149,-0.041]	1.00	43	+/-0.1
Vertebrates	0.03 [0.03]	-0.019, 0.089 [-0.018,0.093]	0.89	100	+/-0.1

Table S8a Model summaries explaining soil bacteria and fungi richness as an interaction of forest developmental stage and elevation for each taxon. Metrics are explained in table S4. MAP and MAP_CI values are on the log-scale, values in brackets are the back-transformed values using the formula *exp(x)-1* and showing the rate of change.

Taxon	Parameter	MAP [trans]	MAP_CI95 [trans]	pd	%inROPE	ROPE	Rhat	ESS
Bacteria	b_Intercept	7.204 [1344.339]	7.115, 7.282 [1229.661,1453.214]	1.00	0	+/-0.1	1	4,306
	b_Establishment	0.04 [0.04]	-0.06, 0.14 [-0.058,0.15]	0.78	90	+/-0.1	1	5,939
	b_Optimum	0.042 [0.043]	-0.06, 0.146 [-0.058,0.157]	0.79	89	+/-0.1	1	5,281
	b_Plenter	0.012 [0.012]	-0.085, 0.115 [-0.082,0.122]	0.61	98	+/-0.1	1	5,793
	b_Terminal	0.047 [0.049]	-0.052, 0.151 [-0.05,0.163]	0.81	87	+/-0.1	1	5,333
	b_elev_z	0.017 [0.017]	-0.064, 0.114 [-0.062,0.12]	0.66	98	+/-0.1	1	2,499
	b_doy_z	0 [0]	-0.041, 0.039 [-0.04,0.04]	0.50	100	+/-0.1	1	7,057
	b_Establishment:elev_z	0.024 [0.025]	-0.091, 0.117 [-0.087,0.125]	0.63	96	+/-0.1	1	3,970
	b_Optimum:elev_z	-0.061 [-0.059]	-0.167, 0.046 [-0.154,0.047]	0.87	77	+/-0.1	1	3,254
	b_Plenter:elev_z	0.002 [0.002]	-0.107, 0.102 [-0.102,0.107]	0.52	99	+/-0.1	1	3,486
	b_Terminal:elev_z	-0.077 [-0.074]	-0.179, 0.025 [-0.164,0.025]	0.93	68	+/-0.1	1	3,736
Fungi	b_Intercept	6.412 [608.368]	6.292, 6.521 [539.006,678.023]	1.00	0	+/-0.1	1	3,940
	b_Establishment	-0.042 [-0.041]	-0.176, 0.108 [-0.161,0.115]	0.68	83	+/-0.1	1	5,252
	b_Optimum	-0.237 [-0.211]	-0.381, -0.096 [-0.317,-0.091]	1.00	1	+/-0.1	1	5,164
	b_Plenter	-0.227 [-0.203]	-0.355, -0.077 [-0.299,-0.074]	1.00	3	+/-0.1	1	4,725
	b_Terminal	-0.107 [-0.101]	-0.244, 0.033 [-0.216,0.034]	0.93	48	+/-0.1	1	5,412
	b_elev_z	0.002 [0.002]	-0.114, 0.113 [-0.108,0.12]	0.51	96	+/-0.1	1	3,151
	b_doy_z	-0.029 [-0.028]	-0.09, 0.022 [-0.086,0.022]	0.89	100	+/-0.1	1	6,409
	b_Establishment:elev_z	0.001 [0.001]	-0.147, 0.149 [-0.137,0.161]	0.51	86	+/-0.1	1	4,475
	b_Optimum:elev_z	0.004 [0.004]	-0.139, 0.145 [-0.13,0.156]	0.51	88	+/-0.1	1	4,160
	b_Plenter:elev_z	-0.005 [-0.005]	-0.154, 0.131 [-0.143,0.14]	0.56	86	+/-0.1	1	4,354
	b_Terminal:elev_z	-0.064 [-0.062]	-0.207, 0.072 [-0.187,0.075]	0.81	71	+/-0.1	1	4,148

Table S8b Model summaries explaining plant and arthropods$_{TAX}$ richness as an interaction of forest developmental stage and elevation for each taxon. Metrics are explained in table S4. MAP and MAP_CI values are on the log-scale, values in brackets are the back-transformed values using the formula *exp(x)-1* and showing the rate of change. Delta values at the bottom of Arthropods$_{TAX}$ are additionally calculated differences of the posterior distributions describing the rate of change between the establishment and terminal stage.

Taxon	Parameter	MAP [trans]	MAP_CI95 [trans]	pd	%inROPE	ROPE	Rhat	ESS
Plants	b_Intercept	4.038 [55.704]	3.926, 4.141 [49.717,61.892]	1.00	0	+/-0.1	1	4,987
	b_Establishment	-0.177 [-0.162]	-0.311, -0.057 [-0.267,-0.056]	1.00	6	+/-0.1	1	6,500
	b_Optimum	-0.199 [-0.18]	-0.32, -0.061 [-0.274,-0.059]	1.00	7	+/-0.1	1	6,860
	b_Plenter	-0.112 [-0.106]	-0.225, 0.027 [-0.202,0.027]	0.95	46	+/-0.1	1	6,814
	b_Terminal	-0.014 [-0.014]	-0.138, 0.115 [-0.129,0.121]	0.59	92	+/-0.1	1	7,194
	b_elev_z	0.012 [0.012]	-0.103, 0.113 [-0.098,0.119]	0.54	98	+/-0.1	1	3,942
	b_doy_z	0.063 [0.065]	0.011, 0.121 [0.011,0.128]	0.99	90	+/-0.1	1	8,546
	b_Establishment:elev_z	0.09 [0.094]	-0.047, 0.224 [-0.046,0.251]	0.90	56	+/-0.1	1	5,299
	b_Optimum:elev_z	0.016 [0.017]	-0.112, 0.148 [-0.106,0.159]	0.61	89	+/-0.1	1	5,509
	b_Plenter:elev_z	0.083 [0.087]	-0.043, 0.213 [-0.042,0.237]	0.90	59	+/-0.1	1	5,025
	b_Terminal:elev_z	0.037 [0.038]	-0.088, 0.163 [-0.084,0.177]	0.69	87	+/-0.1	1	4,784
Arthropods$_{TAX}$	b_Intercept	4.639 [102.394]	4.557, 4.738 [94.319,113.255]	1.00	0	+/-0.1	1	2,973
	b_Establishment	-0.104 [-0.099]	-0.192, -0.011 [-0.174,-0.011]	0.98	48	+/-0.1	1	4,990
	b_Optimum	-0.019 [-0.019]	-0.121, 0.062 [-0.114,0.064]	0.69	97	+/-0.1	1	5,019
	b_Plenter	-0.033 [-0.032]	-0.115, 0.063 [-0.108,0.065]	0.70	98	+/-0.1	1	4,802
	b_Terminal	0.033 [0.034]	-0.058, 0.12 [-0.056,0.128]	0.77	95	+/-0.1	1	4,613
	b_elev_z	-0.188 [-0.172]	-0.269, -0.104 [-0.236,-0.099]	1.00	0	+/-0.1	1	2,989
	b_Establishment:elev_z	0.04 [0.041]	-0.062, 0.129 [-0.06,0.138]	0.78	92	+/-0.1	1	4,167
	b_Optimum:elev_z	0.014 [0.014]	-0.087, 0.095 [-0.083,0.1]	0.56	100	+/-0.1	1	3,832
	b_Plenter:elev_z	-0.001 [-0.001]	-0.094, 0.094 [-0.089,0.099]	0.50	100	+/-0.1	1	3,826
	b_Terminal:elev_z	0.008 [0.008]	-0.073, 0.109 [-0.071,0.115]	0.65	98	+/-0.1	1	4,021
	Delta(E-T)	-0.137 [-0.128]	-0.224, -0.045 [-0.201,-0.044]	1.00	21	+/-0.1		

Table S8c Model summaries explaining $insects_{BIN}$ and vertebrate richness as an interaction of forest developmental stage and elevation for each taxon. Metrics are explained in Table S4. MAP and MAP_CI values are on the log-scale, values in brackets are the back-transformed values using the formula *exp(x)-1* and showing the rate of change.

Taxon	Parameter	MAP [trans]	MAP_CI95 [trans]	pd	%inROPE	ROPE	Rhat	ESS
$Insects_{BIN}$	b_Intercept	7.158 [1283.118]	7.059, 7.263 [1161.885,1425.868]	1.00	0	+/-0.1	1	3,471
	b_Establishment	-0.271 [-0.238]	-0.378, -0.17 [-0.315,-0.156]	1.00	0	+/-0.1	1	6,046
	b_Optimum	-0.334 [-0.284]	-0.449, -0.236 [-0.362,-0.21]	1.00	0	+/-0.1	1	5,645
	b_Plenter	-0.314 [-0.269]	-0.413, -0.203 [-0.338,-0.184]	1.00	0	+/-0.1	1	5,710
	b_Terminal	-0.211 [-0.19]	-0.318, -0.11 [-0.272,-0.104]	1.00	0	+/-0.1	1	6,069
	b_elev_z	-0.158 [-0.146]	-0.248, -0.055 [-0.22,-0.054]	1.00	11	+/-0.1	1	3,277
	b_Establishment:elev_z	0.163 [0.177]	0.056, 0.275 [0.058,0.317]	1.00	10	+/-0.1	1	5,056
	b_Optimum:elev_z	0.034 [0.034]	-0.08, 0.137 [-0.077,0.147]	0.73	92	+/-0.1	1	4,685
	b_Plenter:elev_z	0.03 [0.031]	-0.082, 0.139 [-0.079,0.149]	0.70	92	+/-0.1	1	4,861
	b_Terminal:elev_z	0.026 [0.026]	-0.081, 0.131 [-0.078,0.14]	0.67	94	+/-0.1	1	4,925
Vertebrates	b_Intercept	3.36 [27.787]	3.259, 3.444 [25.023,30.311]	1.00	0	+/-0.1	1	3,523
	b_Establishment	-0.025 [-0.025]	-0.123, 0.086 [-0.116,0.089]	0.64	96	+/-0.1	1	5,933
	b_Optimum	-0.077 [-0.074]	-0.171, 0.042 [-0.157,0.043]	0.89	73	+/-0.1	1	5,651
	b_Plenter	-0.081 [-0.078]	-0.174, 0.041 [-0.16,0.042]	0.89	72	+/-0.1	1	6,090
	b_Terminal	-0.01 [-0.01]	-0.112, 0.099 [-0.106,0.104]	0.53	98	+/-0.1	1	5,681
	b_elev_z	0.015 [0.015]	-0.075, 0.106 [-0.073,0.112]	0.63	99	+/-0.1	1	3,798
	b_Establishment:elev_z	0.035 [0.035]	-0.076, 0.143 [-0.073,0.154]	0.71	91	+/-0.1	1	5,228
	b_Optimum:elev_z	0.029 [0.03]	-0.082, 0.136 [-0.078,0.146]	0.71	91	+/-0.1	1	4,991
	b_Plenter:elev_z	0.026 [0.026]	-0.089, 0.127 [-0.085,0.136]	0.63	95	+/-0.1	1	5,160
	b_Terminal:elev_z	0.013 [0.013]	-0.098, 0.113 [-0.094,0.12]	0.59	98	+/-0.1	1	4,955

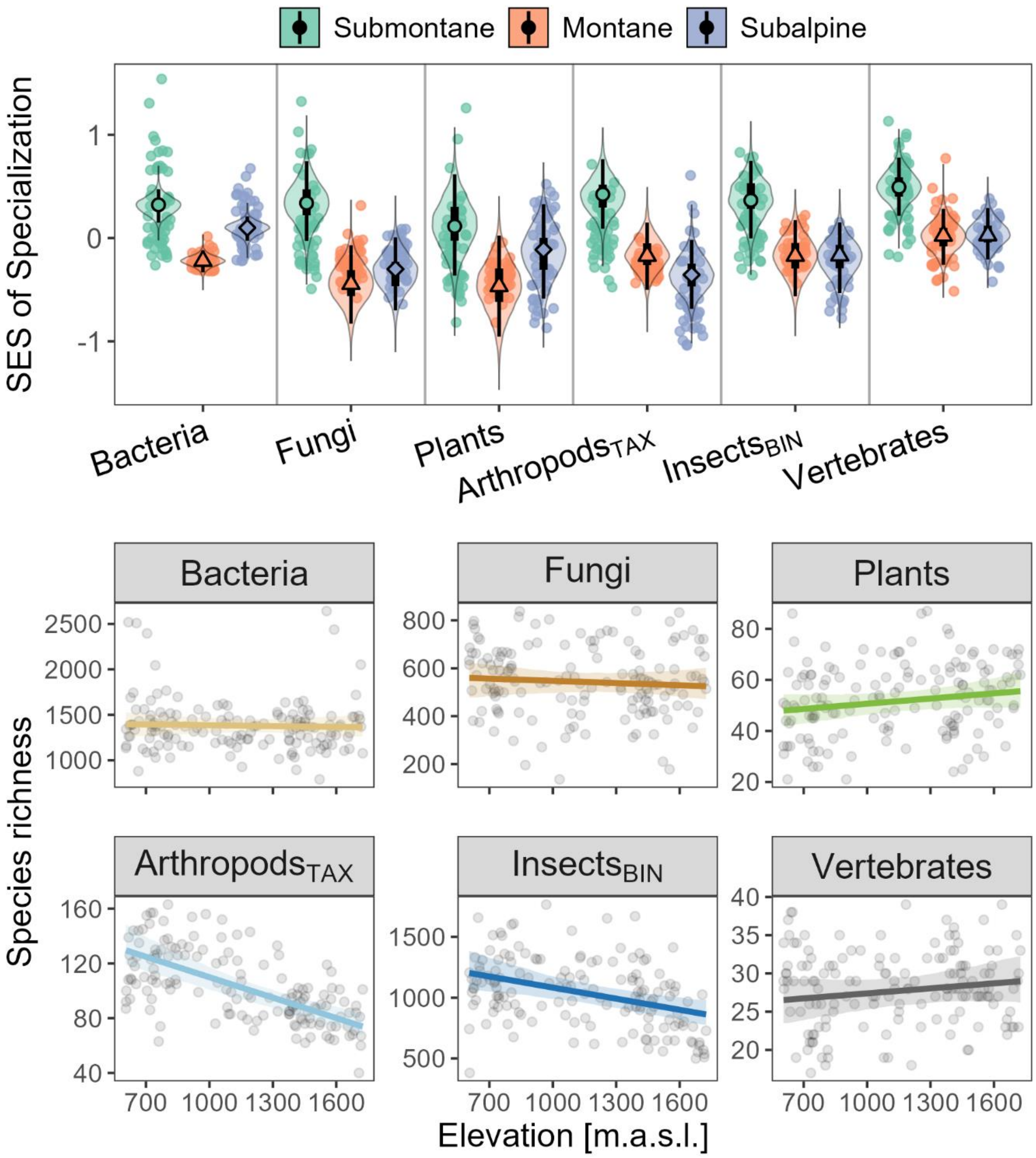

Figure S10 Raw and predicted SES of specialization and species richness along elevation. Trends are the result of predictions along the elevational gradient using individual Bayesian multilevel models for each measure and taxon. We averaged the predictions over forest developmental stages and summarized by means of the MAP, the 95% HDI for both and the 50% HDI (thick bar) for the SES. Different point shapes display substantial differences in SES between elevational zones. We used the minimum and maximum raw values to normalize the predictions for species richness in the range between zero and one for the joint figure including all six taxa in Fig. 3 of the main body.

Table S9 Calculated difference of differences of species richness between each optimum-[developmental stage]-comparison (i.e. Δ O - G, Δ O - E, Δ O - P, Δ O - T) of the montane and subalpine with the submontane zone. Concept is explained in Fig. S6 and differences are visualised in Fig. S10.

Taxon	Comparison	MAP	MAP_CI95	MAP_CI50	pd	%inROPE	ROPE
Bacteria	O-G(Subm-Mont)	54.70	[-65.24,268.33]	[16.09,140.72]	0.85	8	+/-11.07
	O-G(Subm-Suba)	86.05	[-151.44,320.57]	[-25.14,134.23]	0.73	8	+/-11.07
	O-E(Subm-Mont)	111.47	[-107.86,278.93]	[5.51,150.32]	0.79	7	+/-11.07
	O-E(Subm-Suba)	-7.33	[-245.27,252.04]	[-75.72,94.1]	0.51	8	+/-11.07
	O-P(Subm-Mont)	97.34	[-70.81,247.24]	[18.13,134.41]	0.84	8	+/-11.07
	O-P(Subm-Suba)	32.19	[-158.59,276.66]	[-30.92,115.84]	0.69	8	+/-11.07
	O-T(Subm-Mont)	7.85	[-93.92,182.78]	[-22.1,64.23]	0.69	15	+/-11.07
	O-T(Subm-Suba)	1.10	[-182.69,203.14]	[-49.04,65.59]	0.54	12	+/-11.07
Fungi	O-G(Subm-Mont)	0.84	[-89.55,96.53]	[-31.5,30.83]	0.51	11	+/-5.98
	O-G(Subm-Suba)	4.05	[-173.13,184.36]	[-61.26,61.13]	0.51	6	+/-5.98
	O-E(Subm-Mont)	-10.65	[-89.18,94.67]	[-30.85,32.44]	0.50	10	+/-5.98
	O-E(Subm-Suba)	-19.49	[-167.77,184.88]	[-60.03,63.18]	0.51	5	+/-5.98
	O-P(Subm-Mont)	1.06	[-41.48,84.55]	[-10.05,30.26]	0.68	17	+/-5.98
	O-P(Subm-Suba)	2.04	[-87.41,95.56]	[-25.01,30.9]	0.52	13	+/-5.98
	O-T(Subm-Mont)	37.52	[-42.89,128.94]	[11.07,69.75]	0.85	7	+/-5.98
	O-T(Subm-Suba)	67.25	[-72,200.98]	[17.06,110.41]	0.82	4	+/-5.98
Plants	O-G(Subm-Mont)	0.89	[-6.85,8.92]	[-1.75,3.77]	0.60	11	+/-0.54
	O-G(Subm-Suba)	2.09	[-13.45,16.82]	[-3.19,7.68]	0.59	5	+/-0.54
	O-E(Subm-Mont)	0.01	[-4.75,9.3]	[-1.3,3.61]	0.70	13	+/-0.54
	O-E(Subm-Suba)	-0.31	[-11.26,9.06]	[-3.39,3.1]	0.56	10	+/-0.54
	O-P(Subm-Mont)	-1.18	[-7.32,6.79]	[-3.89,0.66]	0.62	12	+/-0.54
	O-P(Subm-Suba)	-5.43	[-15.53,6.19]	[-8.44,-0.92]	0.82	6	+/-0.54
	O-T(Subm-Mont)	-1.23	[-7.99,6.11]	[-3.62,1.27]	0.61	12	+/-0.54
	O-T(Subm-Suba)	-2.62	[-16.17,11.41]	[-6.74,3.17]	0.62	6	+/-0.54
$Arthropods_{TAX}$	O-G(Subm-Mont)	0.82	[-6.19,15.27]	[-1.49,5.45]	0.76	11	+/-0.66
	O-G(Subm-Suba)	0.61	[-9.79,18.96]	[-2.28,6.6]	0.67	9	+/-0.66
	O-E(Subm-Mont)	5.74	[-6.47,18.53]	[1.11,10.1]	0.82	6	+/-0.66
	O-E(Subm-Suba)	9.06	[-10.81,25.57]	[1.84,14.44]	0.81	4	+/-0.66
	O-P(Subm-Mont)	0.68	[-5.9,13.12]	[-1.77,4.49]	0.72	12	+/-0.66
	O-P(Subm-Suba)	0.72	[-9.47,15.28]	[-2.96,4.66]	0.62	10	+/-0.66
	O-T(Subm-Mont)	0.65	[-8.49,13.29]	[-2.93,4.3]	0.62	11	+/-0.66
	O-T(Subm-Suba)	0.58	[-13.6,18.25]	[-4.51,6]	0.56	7	+/-0.66
$Insects_{BIN}$	O-G(Subm-Mont)	108.74	[-65.49,309.12]	[55.93,181.48]	0.90	5	+/-12.13
	O-G(Subm-Suba)	195.93	[-112.49,513.31]	[105.74,314.96]	0.91	3	+/-12.13
	O-E(Subm-Mont)	-5.20	[-135.18,226.84]	[-55.04,92.54]	0.62	9	+/-12.13
	O-E(Subm-Suba)	-96.12	[-279.29,104.2]	[-154.44,-24.11]	0.82	6	+/-12.13
	O-P(Subm-Mont)	6.26	[-60.38,146.26]	[-13.06,51.72]	0.70	22	+/-12.13
	O-P(Subm-Suba)	4.66	[-115.42,164.98]	[-36.61,50.42]	0.60	17	+/-12.13
	O-T(Subm-Mont)	39.75	[-103.39,163.42]	[-17.84,74.34]	0.69	14	+/-12.13
	O-T(Subm-Suba)	70.11	[-165.55,274.06]	[-14.9,139.8]	0.70	8	+/-12.13
Vertebrates	O-G(Subm-Mont)	0.64	[-2.06,4.23]	[-0.35,1.78]	0.73	9	+/-0.19
	O-G(Subm-Suba)	0.26	[-3.8,6.48]	[-0.69,2.86]	0.65	6	+/-0.19
	O-E(Subm-Mont)	0.11	[-2.37,3.31]	[-0.58,1.17]	0.60	12	+/-0.19
	O-E(Subm-Suba)	-0.01	[-4.89,4.55]	[-1.5,1.43]	0.53	8	+/-0.19
	O-P(Subm-Mont)	0.13	[-1.84,3.34]	[-0.42,1.22]	0.66	13	+/-0.19
	O-P(Subm-Suba)	-0.20	[-3.92,3.65]	[-1.25,1.07]	0.52	9	+/-0.19
	O-T(Subm-Mont)	0.24	[-2.16,3.61]	[-0.47,1.39]	0.67	11	+/-0.19
	O-T(Subm-Suba)	0.34	[-4.27,5.46]	[-0.86,2.29]	0.59	7	+/-0.19

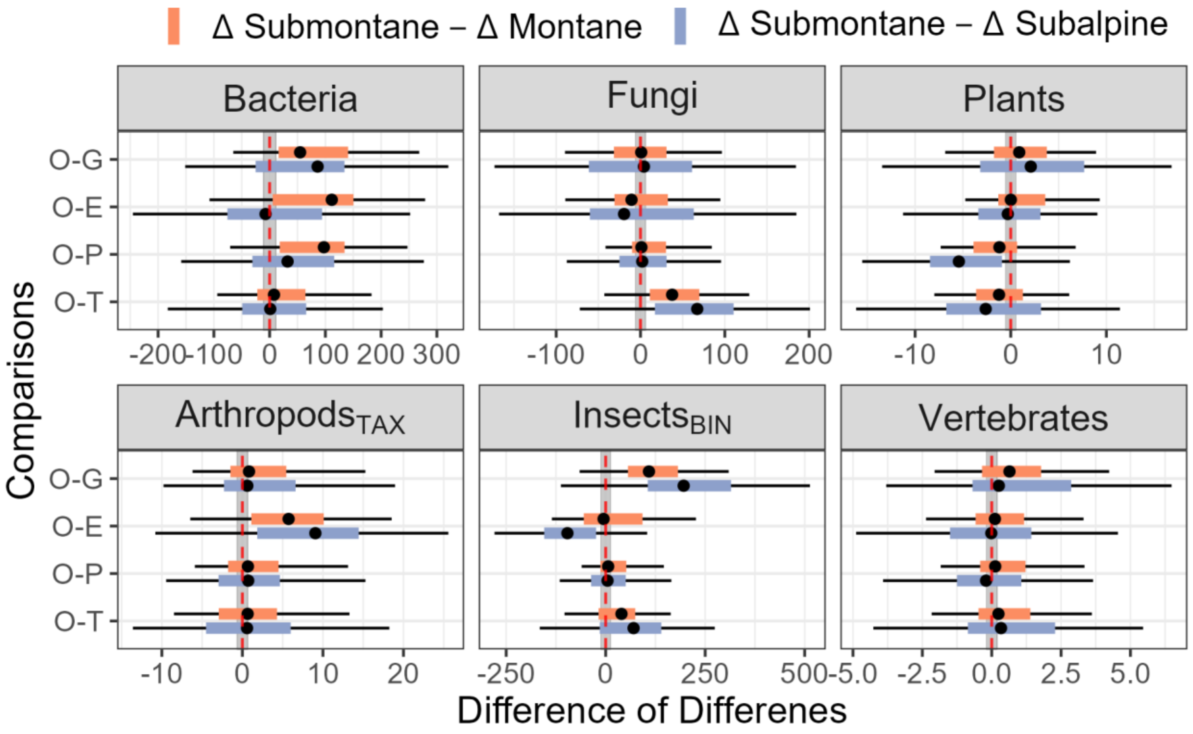

Figure S11 Visualization of the calculated difference of differences in species richness shown in Table S8. Our hypothesis of weaker patterns of species richness with increasing elevation is supported by both positive values and larger differences in the submontane-subalpine compared to the submontane-montane comparison, as shown in Fig. S6. Grey shaded area represents the ROPE.

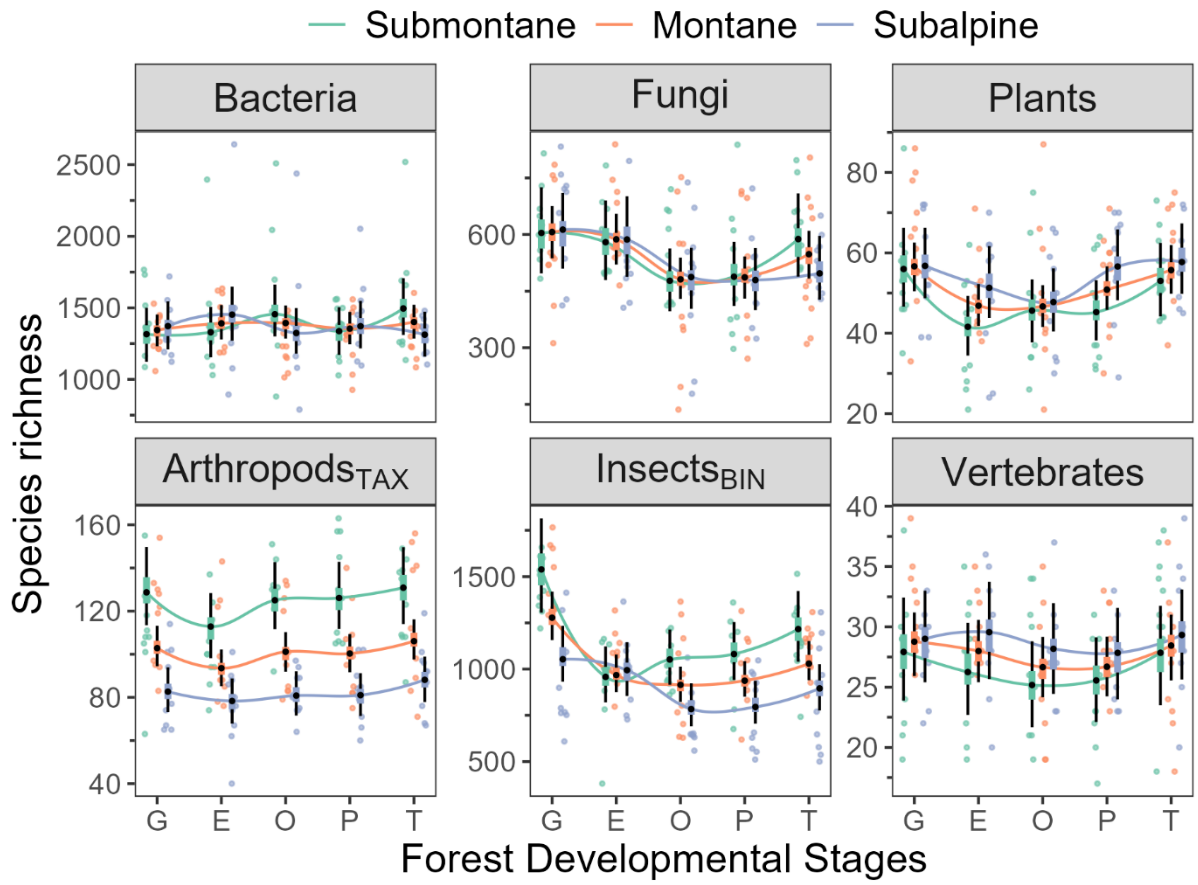

Figure S12 Complementary to Fig. 5 in the main body, patterns of species richness over forest development across elevation belts for each taxonomic group including 50% and 95% HDIs. While trends in Fig. 4 suggest weaker patterns with increasing elevation, Fig. S10 shows that variation in

species richness was high and thus differences between each optimum-[developmental stage]-comparison over the elevational zones (see concept in Fig. S6) did not differ. Difference of differences are provided in Table S10 and Fig. S11.

Jaccard dissimilarity

Table S10 Model summaries explaining Jaccard dissimilarity as a function of elevational zone. Metrics are explained in Table S4.

Taxon	Parameter	MAP	MAP_CI95	pd	%inROPE	ROPE	Rhat	ESS
Bacteria	b_Intercept	0.835	[0.758,0.9]	1.00	0	+/-0.181	1.00	966
	b_elev_class1Montane	-0.194	[-0.292,-0.095]	1.00	42	+/-0.181	1.00	832
	b_elev_class1Subalpine	-0.019	[-0.113,0.086]	0.61	100	+/-0.181	1.00	937
	b_elev_diff_z	-0.001	[-0.01,0.009]	0.56	100	+/-0.181	1.00	12,364
	b_dist_z	0.033	[0.015,0.053]	1.00	100	+/-0.181	1.00	2,003
	b_doy_diff_z	0.014	[0.005,0.022]	1.00	100	+/-0.181	1.00	12,401
Fungi	b_Intercept	1.644	[1.574,1.721]	1.00	0	+/-0.181	1.00	500
	b_elev_class1Montane	-0.030	[-0.131,0.077]	0.73	100	+/-0.181	1.00	543
	b_elev_class1Subalpine	-0.031	[-0.131,0.078]	0.71	100	+/-0.181	1.01	530
	b_elev_diff_z	0.024	[0.016,0.032]	1.00	100	+/-0.181	1.00	8,008
	b_polydist_z21	2.051	[1.089,3.063]	1.00	0	+/-0.181	1.01	734
	b_polydist_z22	-1.955	[-2.428,-1.542]	1.00	0	+/-0.181	1.00	4,889
	b_doy_diff_z	0.025	[0.017,0.032]	1.00	100	+/-0.181	1.00	7,838
Plants	b_Intercept	0.836	[0.754,0.901]	1.00	0	+/-0.181	1.00	1,193
	b_elev_class1Montane	-0.188	[-0.283,-0.086]	1.00	46	+/-0.181	1.00	1,128
	b_elev_class1Subalpine	-0.018	[-0.115,0.081]	0.66	100	+/-0.181	1.00	1,341
	b_elev_diff_z	-0.001	[-0.011,0.009]	0.59	100	+/-0.181	1.00	14,530
	b_dist_z	0.036	[0.015,0.054]	1.00	100	+/-0.181	1.00	1,852
	b_doy_diff_z	0.011	[0.001,0.02]	0.98	100	+/-0.181	1.00	11,822
$Arthropods_{TAX}$	b_Intercept	0.895	[0.836,0.96]	1.00	0	+/-0.181	1.01	1,061
	b_elev_class1Montane	0.183	[0.102,0.27]	1.00	48	+/-0.181	1.01	998
	b_elev_class1Subalpine	0.221	[0.136,0.304]	1.00	16	+/-0.181	1.00	1,249
	b_elev_diff_z	0.115	[0.106,0.124]	1.00	100	+/-0.181	1.00	12,263
	b_polydist_z21	5.617	[4.726,6.582]	1.00	0	+/-0.181	1.00	2,059
	b_polydist_z22	-3.006	[-3.479,-2.555]	1.00	0	+/-0.181	1.00	8,608
$Insects_{BIN}$	b_Intercept	1.010	[0.951,1.063]	1.00	0	+/-0.181	1.00	585
	b_elev_class1Montane	-0.062	[-0.142,0.02]	0.93	100	+/-0.181	1.00	698
	b_elev_class1Subalpine	-0.066	[-0.144,0.014]	0.95	100	+/-0.181	1.00	767
	b_elev_diff_z	0.064	[0.059,0.07]	1.00	100	+/-0.181	1.00	11,703
	b_polydist_z21	4.303	[3.47,4.987]	1.00	0	+/-0.181	1.00	1,100
	b_polydist_z22	-2.286	[-2.568,-1.964]	1.00	0	+/-0.181	1.00	7,307
Vertebrates	b_Intercept	0.304	[0.222,0.389]	1.00	0	+/-0.181	1.01	1,064
	b_elev_class1Montane	-0.217	[-0.33,-0.107]	1.00	25	+/-0.181	1.00	1,437
	b_elev_class1Subalpine	-0.275	[-0.38,-0.157]	1.00	4	+/-0.181	1.00	1,155
	b_elev_diff_z	0.062	[0.049,0.073]	1.00	100	+/-0.181	1.00	12,720
	b_polydist_z21	3.597	[2.382,4.695]	1.00	0	+/-0.181	1.00	2,115
	b_polydist_z22	-4.930	[-5.534,-4.303]	1.00	0	+/-0.181	1.00	9,996

Table S11 Calculated differences of the Jaccard dissimilarity between forest developmental stages across the elevational zones. Metrics are explained in Table S4.

Taxon	Comparison	MAP	MAP_CI95	MAP_CI50	pd	%inROPE	ROPE
Bacteria	Submontane - Montane	0.042	[0.02,0.063]	[0.035,0.05]	1.00	0	+/-0.003
	Submontane - Subalpine	0.004	[-0.018,0.024]	[-0.004,0.01]	0.61	25	+/-0.003
	Montane - Subalpine	-0.039	[-0.061,-0.018]	[-0.046,-0.032]	1.00	0	+/-0.003
Fungi	Submontane - Montane	0.004	[-0.009,0.019]	[-0.001,0.009]	0.73	7	+/-0.001
	Submontane - Subalpine	0.004	[-0.01,0.018]	[0,0.009]	0.71	8	+/-0.001
	Montane - Subalpine	0.000	[-0.015,0.013]	[-0.005,0.004]	0.55	9	+/-0.001
Plants	Submontane - Montane	0.041	[0.019,0.062]	[0.033,0.048]	1.00	0	+/-0.003
	Submontane - Subalpine	0.004	[-0.017,0.025]	[-0.003,0.012]	0.66	25	+/-0.003
	Montane - Subalpine	-0.034	[-0.058,-0.016]	[-0.044,-0.029]	1.00	0	+/-0.003
$Arthropods_{TAX}$	Submontane - Montane	-0.034	[-0.053,-0.02]	[-0.041,-0.029]	1.00	0	+/-0.002
	Submontane - Subalpine	-0.043	[-0.059,-0.027]	[-0.048,-0.037]	1.00	0	+/-0.002
	Montane - Subalpine	-0.006	[-0.023,0.009]	[-0.012,-0.002]	0.80	12	+/-0.002
$Insects_{BIN}$	Submontane - Montane	0.012	[-0.004,0.028]	[0.007,0.018]	0.93	3	+/-0.001
	Submontane - Subalpine	0.013	[-0.003,0.028]	[0.008,0.019]	0.95	3	+/-0.001
	Montane - Subalpine	0.002	[-0.016,0.017]	[-0.004,0.007]	0.55	10	+/-0.001
Vertebrates	Submontane - Montane	0.053	[0.026,0.081]	[0.045,0.064]	1.00	0	+/-0.003
	Submontane - Subalpine	0.068	[0.039,0.094]	[0.056,0.075]	1.00	0	+/-0.003
	Montane - Subalpine	0.012	[-0.015,0.039]	[0.003,0.022]	0.82	11	+/-0.003

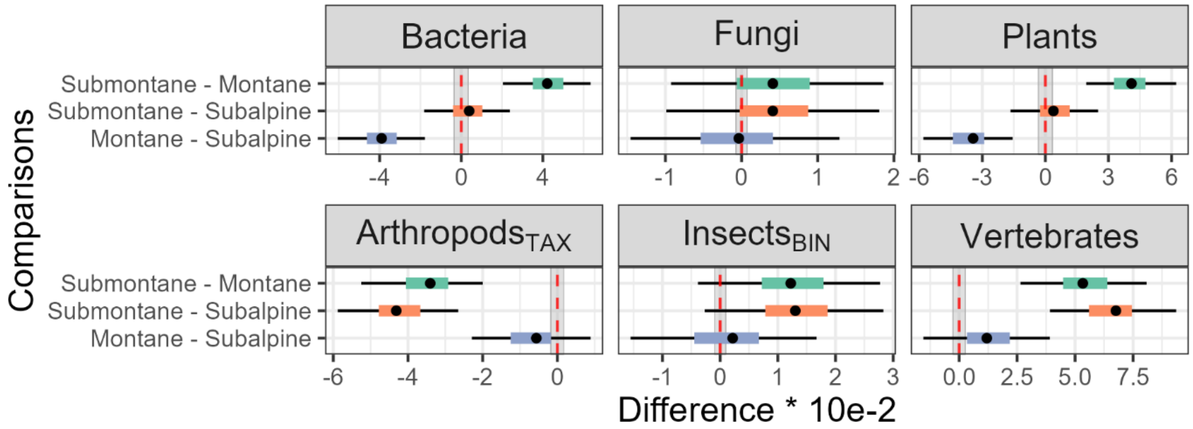

Figure S13 Visualization of the calculated differences of the Jaccard dissimilarity shown in Table S10. Points show the MAP and bars the 50% (thick coloured bars) and 95% HDIs. Grey shaded area represents the ROPE.

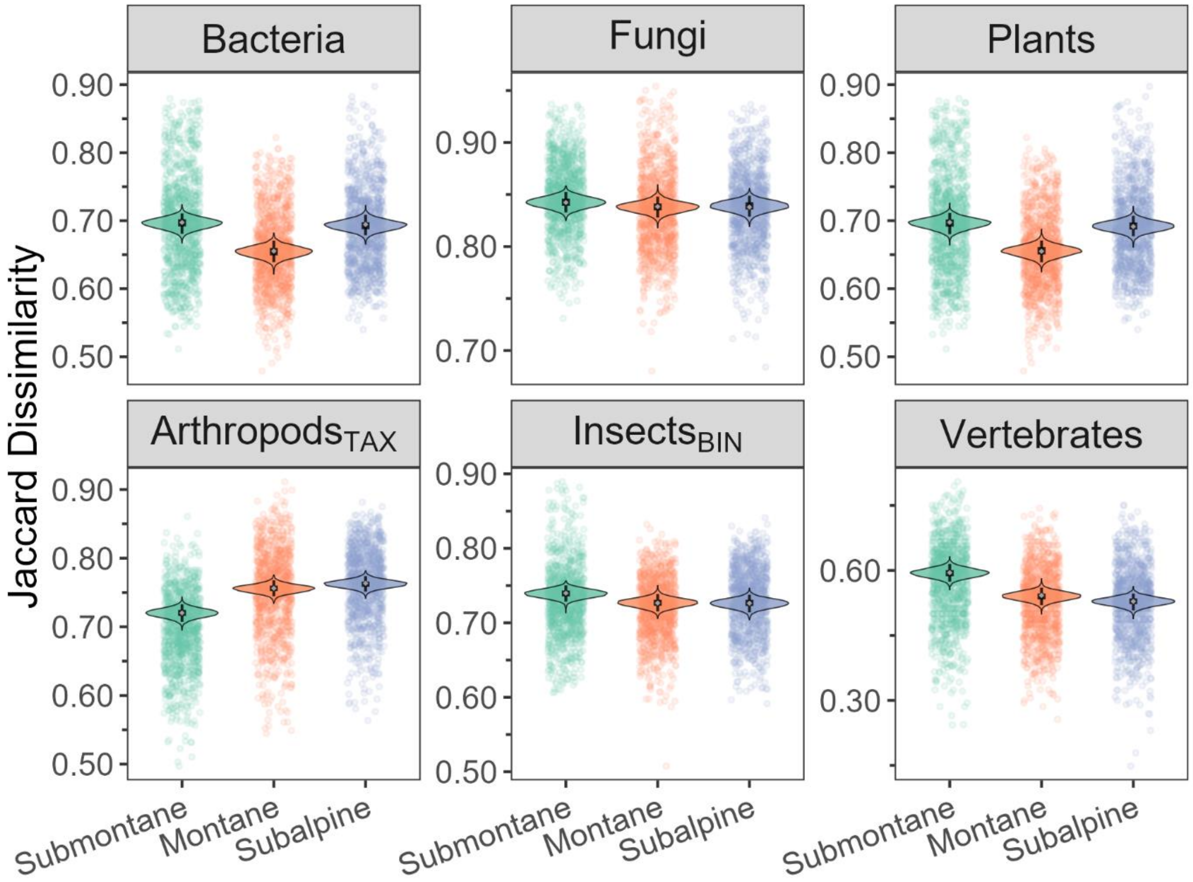

Figure S14 Complementary to Fig. 4 in the main body, Jaccard dissimilarity for each taxonomic group across the three elevational zones including data points. Posterior distributions were summarized by means of the MAP (grey point) and 95% and 50% (thick bars) HDI.

Canopy Cover

Table S12 Model summaries explaining canopy cover by an interaction of forest developmental stage and elevation. Metrics are explained in Table S4.

Parameter	MAP	MAP_CI95	pd	%inROPE	ROPE	Rhat	ESS
b_Intercept	-1.29	[-1.66,-0.92]	1.00	0	+/-0.18	1	3,569
b_Establishment	1.01	[0.64,1.42]	1.00	0	+/-0.18	1	5,005
b_Optimum	1.77	[1.37,2.18]	1.00	0	+/-0.18	1	4,442
b_Plenter	1.86	[1.46,2.26]	1.00	0	+/-0.18	1	4,826
b_Terminal	1.74	[1.28,2.08]	1.00	0	+/-0.18	1	4,702
b_elev_z	-0.22	[-0.61,0.13]	0.88	41	+/-0.18	1	2,939
b_Establishment:elev_z	-0.71	[-1.16,-0.3]	1.00	0	+/-0.18	1	4,422
b_Optimum:elev_z	-0.43	[-0.9,-0.04]	0.98	9	+/-0.18	1	3,879
b_Plenter:elev_z	-0.27	[-0.68,0.17]	0.89	32	+/-0.18	1	4,052
b_Terminal:elev_z	-0.16	[-0.58,0.26]	0.78	51	+/-0.18	1	3,836

Table S13 Model summaries explaining the PV (Proportional Variability) of the microclimate by an interaction of forest developmental stage and elevation as fourth order polynomial. We calculated the PV[1] for each plot and day from temperature measures of Tomst TMS-4 loggers. Metrics are explained in Table S4.

Parameter	MAP	MAP_CI95	pd	%inROPE	ROPE	Rhat	ESS
b_Intercept	-1.45	[-1.53,-1.36]	1.00	0	+/-0.18	1.01	824
b_Establishment	-0.23	[-0.35,-0.12]	1.00	15	+/-0.18	1.00	1,193
b_Optimum	-0.22	[-0.33,-0.1]	1.00	25	+/-0.18	1.01	1,088
b_Plenter	-0.22	[-0.34,-0.11]	1.00	21	+/-0.18	1.00	1,054
b_Terminal	-0.21	[-0.33,-0.09]	1.00	31	+/-0.18	1.00	1,000
b_polyelev_z41	4.22	[0.57,7.57]	0.99	0	+/-0.18	1.00	3,273
b_polyelev_z42	0.50	[-2.97,3.94]	0.61	8	+/-0.18	1.00	3,229
b_polyelev_z43	0.52	[-3.15,3.96]	0.57	8	+/-0.18	1.00	3,422
b_polyelev_z44	0.61	[-3.18,3.61]	0.59	8	+/-0.18	1.00	2,869
b_Establishment:polyelev_z41	15.53	[5.33,24.52]	1.00	0	+/-0.18	1.00	2,028
b_Optimum:polyelev_z41	9.21	[0.42,19.35]	0.97	0	+/-0.18	1.00	1,817
b_Plenter:polyelev_z41	9.25	[-0.03,19.53]	0.97	0	+/-0.18	1.00	2,278
b_Terminal:polyelev_z41	15.50	[4.51,24.7]	1.00	0	+/-0.18	1.00	1,923
b_Establishment:polyelev_z42	1.17	[-8.05,11.1]	0.62	3	+/-0.18	1.00	2,248
b_Optimum:polyelev_z42	0.91	[-8.18,10.8]	0.61	3	+/-0.18	1.00	2,112
b_Plenter:polyelev_z42	2.68	[-6.14,13.53]	0.75	3	+/-0.18	1.00	1,931
b_Terminal:polyelev_z42	-0.54	[-9.5,8.07]	0.56	3	+/-0.18	1.00	1,686
b_Establishment:polyelev_z43	6.78	[-2.52,16.43]	0.91	1	+/-0.18	1.00	2,166
b_Optimum:polyelev_z43	0.24	[-8.6,10.85]	0.55	3	+/-0.18	1.01	2,246
b_Plenter:polyelev_z43	8.32	[-0.43,18.05]	0.97	1	+/-0.18	1.00	1,893
b_Terminal:polyelev_z43	3.75	[-6.1,12.58]	0.78	2	+/-0.18	1.00	2,052
b_Establishment:polyelev_z44	-7.07	[-15.89,2.09]	0.93	1	+/-0.18	1.00	1,999
b_Optimum:polyelev_z44	-5.13	[-14.09,5.95]	0.81	2	+/-0.18	1.00	2,149
b_Plenter:polyelev_z44	6.40	[-4.44,16.16]	0.88	1	+/-0.18	1.00	1,911
b_Terminal:polyelev_z44	3.66	[-7.99,12.16]	0.69	3	+/-0.18	1.00	1,981

References

1. Heath, J. P. Quantifying temporal variability in population abundances. *Oikos* **115,** 573–581; 10.1111/j.2006.0030-1299.15067.x (2006).
2. Dochtermann, N. A. & Peacock, M. M. Differences in population size variability among populations and species of the family Salmonidae. *Journal of Animal Ecology* **79,** 888–896; 10.1111/j.1365-2656.2010.01686.x (2010).
3. Zenner, E. K., Peck, J. E., Hobi, M. L. & Commarmot, B. Validation of a classification protocol: Meeting the prospect requirement and ensuring distinctiveness when assigning forest development phases. *Appl Veg Sci* **19,** 541–552; 10.1111/avsc.12231 (2016).
4. Singh, R. P., Böttger, D. & Brehm, G. Moth light traps perform better with vanes: A comparison of different designs. *J Applied Entomology* **146,** 1343–1352; 10.1111/jen.13068 (2022).
5. Brehm, G. A new LED lamp for the collection of nocturnal Lepidoptera and a spectral comparison of light-trapping lamps. *NL* **40,** 87–108; 10.3897/nl.40.11887 (2017).

6. Hammer, M., Zahn, A. & Marckmann, U. *Kriterien für die Wertung von Artnachweisen basierend auf Lautaufnahmen* (Koordinationsstellen für Fledermausschutz in Bayern, 2009).

7. Bayerisches Landesamt für Umwelt. *Bestimmung von Fledermausrufaufnahmen und Kriterien für die Wertung von akustischen Artnachweisen Teil 1 - Gattungen Nyctalus, Eptesicus, Vespertilio, Pipistrellus (nyctaloide und pipistrelloide Arten), Mopsfledermaus, Langohrfledermäuse und Hufeisennasen Bayerns - Bearbeitung: Burkard Pfeiffer, Ulrich Marckmann* (Augsburg, 2020).

8. Bayerisches Landesamt für Umwelt. *Bestimmung von Fledermausrufaufnahmen und Kriterien für die Wertung von akustischen Artnachweisen - Teil 2 - Gattung Myotis - Bearbeitung: Burkard Pfeiffer, Ulrich Marckmann* (Augsburg, 2022).

9. Skiba, R. *Europäische Fledermäuse - Kennzeichen, Echoortung und Detektoranwendung.* 2nd ed. (Die Neue Brehm-Bücherei Bd. 648, Westarp Wissenschaften, Hohenwarsleben, 2009).

10. Leray, M. *et al.* A new versatile primer set targeting a short fragment of the mitochondrial COI region for metabarcoding metazoan diversity: application for characterizing coral reef fish gut contents. *Front Zool* **10,** 34; 10.1186/1742-9994-10-34 (2013).

11. Geller, J., Meyer, C., Parker, M. & Hawk, H. Redesign of PCR primers for mitochondrial cytochrome c oxidase subunit I for marine invertebrates and application in all-taxa biotic surveys. *Molecular Ecology Resources* **13,** 851–861; 10.1111/1755-0998.12138 (2013).

12. Rognes, T., Flouri, T., Nichols, B., Quince, C. & Mahé, F. VSEARCH: a versatile open source tool for metagenomics. *PeerJ* **4,** e2584; 10.7717/peerj.2584 (2016).

13. Martin, M. Cutadapt removes adapter sequences from high-throughput sequencing reads. *EMBnet j.* **17,** 10; 10.14806/ej.17.1.200 (2011).

14. Edgar, R. C., Haas, B. J., Clemente, J. C., Quince, C. & Knight, R. UCHIME improves sensitivity and speed of chimera detection. *Bioinformatics* **27,** 2194–2200; 10.1093/bioinformatics/btr381 (2011).

15. Edgar, R. C. Search and clustering orders of magnitude faster than BLAST. *Bioinformatics* **26,** 2460–2461; 10.1093/bioinformatics/btq461 (2010).

16. Camacho, C. *et al.* BLAST+: architecture and applications. *BMC Bioinformatics* **10,** 421; 10.1186/1471-2105-10-421 (2009).

17. Hausmann, A. *et al.* Genetic patterns in European geometrid moths revealed by the Barcode Index Number (BIN) system. *PloS one* **8,** e84518; 10.1371/journal.pone.0084518 (2013).

18. Schmidt, S., Schmid-Egger, C., Morinière, J., Haszprunar, G. & Hebert, P. D. N. DNA barcoding largely supports 250 years of classical taxonomy: identifications for Central European bees (Hymenoptera, Apoidea partim). *Molecular Ecology Resources* **15,** 985–1000; 10.1111/1755-0998.12363 (2015).

19. Pentinsaari, M., Hebert, P. D. N. & Mutanen, M. Barcoding beetles: a regional survey of 1872 species reveals high identification success and unusually deep interspecific divergences. *PloS one* **9,** e108651; 10.1371/journal.pone.0108651 (2014).

20. Moll, J. *et al.* Bacteria inhabiting deadwood of 13 tree species are heterogeneously distributed between sapwood and heartwood. *Environmental microbiology* **20,** 3744–3756; 10.1111/1462-2920.14376 (2018).

21. Leonhardt, S. *et al.* Molecular fungal community and its decomposition activity in sapwood and heartwood of 13 temperate European tree species. *PloS one* **14,** e0212120; 10.1371/journal.pone.0212120 (2019).

22. Kattge, J. *et al.* TRY plant trait database - enhanced coverage and open access. *Global change biology* **26,** 119–188; 10.1111/gcb.14904 (2020).

23. Braziunas, K. H. *et al.* Projected climate and canopy change lead to thermophilization and homogenization of forest floor vegetation in a hotspot of plant species richness. *Global change biology* **30,** e17121; 10.1111/gcb.17121 (2024).

24. Glasmann, F., Senf, C., Seidl, R. & Annighöfer, P. Mapping subcanopy light regimes in temperate mountain forests from Airborne Laser Scanning, Sentinel-1 and Sentinel-2. *Science of Remote Sensing* **8,** 100107; 10.1016/j.srs.2023.100107 (2023).

25. Mandl, L., Stritih, A., Seidl, R., Ginzler, C. & Senf, C. Spaceborne LiDAR for characterizing forest structure across scales in the European Alps. *Remote Sens Ecol Conserv* **9,** 599–614; 10.1002/rse2.330 (2023).

26. Roussel, J.-R. *et al.* lidR: An R package for analysis of Airborne Laser Scanning (ALS) data. *Remote Sensing of Environment* **251,** 112061; 10.1016/j.rse.2020.112061 (2020).

27. Wild, J. *et al.* Climate at ecologically relevant scales: A new temperature and soil moisture logger for long-term microclimate measurement. *Agricultural and Forest Meteorology* **268,** 40–47; 10.1016/j.agrformet.2018.12.018 (2019).

28. Heath, J. P. & Borowski, P. Quantifying proportional variability. *PLOS ONE* **8,** e84074; 10.1371/journal.pone.0084074 (2013).

29. Bürkner, P.-C. brms: An R Package for Bayesian Multilevel Models Using Stan. *J. Stat. Soft.* **80,** 1–28; 10.18637/jss.v080.i01 (2017).

30. Dormann, C. F. *et al.* Methods to Account for Spatial Autocorrelation in the Analysis of Species Distributional Data: A Review. *Ecography* **30,** 609–628 (2007).

31. Vehtari, A., Gelman, A., Simpson, D., Carpenter, B. & Bürkner, P.-C. Rank-Normalization, Folding, and Localization: An Improved Rˆ for Assessing Convergence of MCMC (with Discussion). *ba* **16,** 667–718; 10.1214/20-BA1221 (2021).

32. Gelman, A., Hill, J. & Vehtari, A. *Regression and other stories* (Cambridge University Press, Cambridge, New York, NY, Port Melbourne, VIC, New Delhi, Singapore, 2021).

33. McElreath, R. *Statistical Rethinking, 2nd Edition. A Bayesian Course with Examples in R and STAN.* 2nd ed. (Chapman and Hall/CRC; Safari, Erscheinungsort nicht ermittelbar, Boston, MA, 2020).

34. Hartig, F. *DHARMa: Residual Diagnostics for Hierarchical (Multi-Level / Mixed) Regression Models. R package version 0.4.6* (2022).

35. Gabry, J. & Mahr, T. *Bayesplot: plotting for Bayesian models.* (2022).

36. Lüdecke, D., Ben-Shachar, M. S., Patil, I., Waggoner, P. & Makowski, D. *performance: An R Package for Assessment, Comparison and Testing of Statistical Models* (Center for Open Science, 2021).
