# Supplementary material for: Effects of climate and forest development on habitat specialization and biodiversity in Central European mountain forests
Source: Commun Biol. 2024 Nov 15;7:1518. doi: 10.1038/s42003-024-07239-6 (PMC11568152; doi:10.1038/s42003-024-07239-6)
Supplement: Supplementary file 3 — Reporting Summary [file 42003_2024_7239_MOESM3_ESM.pdf]

nature portfolio

Corresponding author(s): Tobias Richter

Last updated by author(s): Nov 6, 2024

Reporting Summary

Nature Portfolio wishes to improve the reproducibility of the work that we publish. This form provides structure for consistency and transparency in reporting. For further information on Nature Portfolio policies, see our Editorial Policies and the Editorial Policy Checklist.

Statistics

For all statistical analyses, confirm that the following items are present in the figure legend, table legend, main text, or Methods section.

n/a	Confirmed	
☐	☒	The exact sample size (*n*) for each experimental group/condition, given as a discrete number and unit of measurement
☐	☒	A statement on whether measurements were taken from distinct samples or whether the same sample was measured repeatedly
☐	☒	The statistical test(s) used AND whether they are one- or two-sided<br>*Only common tests should be described solely by name; describe more complex techniques in the Methods section.*
☐	☒	A description of all covariates tested
☐	☒	A description of any assumptions or corrections, such as tests of normality and adjustment for multiple comparisons
☐	☒	A full description of the statistical parameters including central tendency (e.g. means) or other basic estimates (e.g. regression coefficient) AND variation (e.g. standard deviation) or associated estimates of uncertainty (e.g. confidence intervals)
☒	☐	For null hypothesis testing, the test statistic (e.g. *F*, *t*, *r*) with confidence intervals, effect sizes, degrees of freedom and *P* value noted<br>*Give P values as exact values whenever suitable.*
☐	☒	For Bayesian analysis, information on the choice of priors and Markov chain Monte Carlo settings
☐	☒	For hierarchical and complex designs, identification of the appropriate level for tests and full reporting of outcomes
☒	☐	Estimates of effect sizes (e.g. Cohen's *d*, Pearson's *r*), indicating how they were calculated

Our web collection on statistics for biologists contains articles on many of the points above.

Software and code

Policy information about availability of computer code

Data collection	No code used for data collection
Data analysis	All analysis were conducted in R, and the code has been deposited in the same Dryad archive as the data: doi:10.5061/dryad.bk3j9kdkp

For manuscripts utilizing custom algorithms or software that are central to the research but not yet described in published literature, software must be made available to editors and reviewers. We strongly encourage code deposition in a community repository (e.g. GitHub). See the Nature Portfolio guidelines for submitting code & software for further information.

Data

Policy information about availability of data

All manuscripts must include a data availability statement. This statement should provide the following information, where applicable:

- Accession codes, unique identifiers, or web links for publicly available datasets
- A description of any restrictions on data availability
- For clinical datasets or third party data, please ensure that the statement adheres to our policy

All data used in our analysis have been deposited in a publicly accessible archive on Dryad: doi:10.5061/dryad.bk3j9kdkp

Human research participants

Policy information about studies involving human research participants and Sex and Gender in Research.

Reporting on sex and gender	Human research participants were not involved in the study.
Population characteristics	Human research participants were not involved in the study.
Recruitment	Human research participants were not involved in the study.
Ethics oversight	Human research participants were not involved in the study.

Note that full information on the approval of the study protocol must also be provided in the manuscript.

Field-specific reporting

Please select the one below that is the best fit for your research. If you are not sure, read the appropriate sections before making your selection.

☐ Life sciences ☐ Behavioural & social sciences ☒ Ecological, evolutionary & environmental sciences

For a reference copy of the document with all sections, see nature.com/documents/nr-reporting-summary-flat.pdf

Ecological, evolutionary & environmental sciences study design

All studies must disclose on these points even when the disclosure is negative.

Study description	We tested the interactive effect of elevation and forest development after disturbance on habitat specialization, species richness, and beta diversity of six taxonomic groups based on assumptions derived from the altitudinal-niche-breadth hypothesis.
Research sample	We sampled six taxonomic groups: bacteria, fungi, plants, arthropods identified by taxonomists, insects identified through DNA metabarcoding, and vertebrates.
Sampling strategy	We selected plots covering five forest developmental stages (gap, establishment, optimum, plenter, terminal) replicated ten times each across three elevational zones (submontane, montane, subalpine), resulting in 150 plots in total.
Data collection	We sampled bacteria and fungi using soil samples and DNA metabarcoding for species identification, plants were sampled on a 200 m2 quadratic area around the plot centre, arthropods identified by taxonomists were sampled using pitfall and light traps, insects identified through DNA metabarcoding were sampled using Malaise traps, and vertebrates were sampled using audio recorder (bats and birds), wild cameras (large mammals), and pitfall traps (small mammals).
Timing and spatial scale	Samplings were conducted between end of April and mid September within Berchtesgaden National Park
Data exclusions	No data was excluded
Reproducibility	We used the reciprocal of the Simpson index to determine habitat diversity based on a species´ proportional use of forest developmental stages, separately for each elevational zone. We then used a null model approach which allows to account for the increasing probability of using more developmental stages with more occupancies. To do so, we simulated 500 random communities and calculated a standardized effect size (SES) for each species and elevational zone by computing habitat diversity for each simulated community, subtracting the mean simulated from the observed habitat diversity, and dividing by the standard deviation of the simulated habitat diversity and multiplied the SES by -1 to obtain an index for specialization and averaged across species of each plot and elevational zone. We calculated species richness as the sum of all species found at a study plot. To quantify beta diversity, we calculated the Jaccard dissimilarity between each set of two plots from different forest developmental stages for each elevational zone, respectively.
Randomization	Randomization was not relevant due to our stratified sampling design.
Blinding	Blinding was not relevant due to our stratified sampling design.

Did the study involve field work? ☒ Yes ☐ No

Field work, collection and transport

Field conditions	Pitfall traps, Malaise traps, and wildcameras were permanently active, light traps and audio recorder only during suitable weather conditions without rain and strong winds.
Location	This study was conducted at Berchtesgaden National Park located in the northern limestone Alps of south-east Germany and study plots were located between 605 m and 1725 m asl.
Access & import/export	We accessed study plots by car and by foot. All cars used for field work holded a permit to access the national park issued by the district and national park administration. Berchtesgaden National Park holds a special permit for sampling arthropods for scientific purposes, which was issued by the regional government of Upper Bavaria.
Disturbance	Disturbance was minimized by staying on official paths and trails as close as possible to the study plots.

Reporting for specific materials, systems and methods

We require information from authors about some types of materials, experimental systems and methods used in many studies. Here, indicate whether each material, system or method listed is relevant to your study. If you are not sure if a list item applies to your research, read the appropriate section before selecting a response.

Materials & experimental systems

n/a	Involved in the study
☒	☐ Antibodies
☒	☐ Eukaryotic cell lines
☒	☐ Palaeontology and archaeology
☐	☒ Animals and other organisms
☒	☐ Clinical data
☒	☐ Dual use research of concern

Methods

n/a	Involved in the study
☒	☐ ChIP-seq
☒	☐ Flow cytometry
☒	☐ MRI-based neuroimaging

Animals and other research organisms

Policy information about studies involving animals; ARRIVE guidelines recommended for reporting animal research, and Sex and Gender in Research

Laboratory animals	No Laboratory animals involved in the study.
Wild animals	We captured arthropods using pitfall, light, and Malaise traps and used copper sulfate (pitfall traps), chlorophorm (light traps), and ethanol (Malaise traps) as killing agents. Arthdopods had to be killed for species identification.
Reporting on sex	This information has not been collected
Field-collected samples	Pitfall and Malaise traps samples were stored in 70% ethanol under cool and dark conditions. Samples from light traps were stored in a freezer.
Ethics oversight	Berchtesgaden National Park holds a special permit for sampling arthropods for scientific purposes, which was issued by the regional government of Upper Bavaria.

Note that full information on the approval of the study protocol must also be provided in the manuscript.
